# High‐Throughput DNA Barcoding Reveals Multi‐Trophic Networks of Plants, Leafminers and Parasitoids in a Tropical Dry Forest

**DOI:** 10.1111/1755-0998.70183

**Published:** 2026-08-26

**Authors:** Ariel H. Muñoz‐Sánchez, Gonzalo Contreras‐Negrete, Alejandro Zaldívar‐Riverón, Rafael Torres Colín, Evgeny V. Zakharov, Carlos López‐Vaamonde, Antonio Hernández‐López

**Affiliations:** ^1^ Ciencias Agrogenómicas, Escuela Nacional de Estudios Superiores Unidad León Universidad Nacional Autónoma de México León Guanajuato Mexico; ^2^ Posgrado en Ciencias Biológicas Universidad Nacional Autónoma de México Mexico City Mexico; ^3^ Colección Nacional de Insectos, Instituto de Biología Universidad Nacional Autónoma de México, Cd. Universitaria Ciudad de México Mexico; ^4^ Herbario Nacional, Instituto de Biología Universidad Nacional Autónoma de México, Cd. Universitaria Ciudad de México Mexico; ^5^ Canadian Centre for DNA Barcoding, Centre for Biodiversity Genomics University of Guelph Guelph Ontario Canada; ^6^ Department of Integrative Biology University of Guelph Guelph Ontario Canada; ^7^ Institut de Recherche Sur la Biologie de L'insecte, CNRS UMR 7261, Université de Tours, UFR Sciences et Techniques Tours France; ^8^ INRAE, UR633, Zoologie Forestière Orléans France; ^9^ 2nd Zoological Department Natural History Museum Vienna Vienna Austria

## Abstract

Tropical dry forests host highly diverse insect communities and complex trophic interactions, yet these networks remain difficult to resolve using conventional molecular or rearing‐based approaches. Here, we integrate long‐read DNA barcoding, non‐target sequence co‐amplification, and host plant taxonomy to reconstruct tri‐trophic interactions among leaf‐mining insects, their host plants, and associated parasitoids in a Mexican tropical dry forest. Using Single Molecule, Real‐Time (SMRT) sequencing on the PacBio Sequel IIe platform, we generated high‐fidelity barcodes from individual leaf‐miner larvae and from parasitoid larvae and pupae recovered from leaf mines. From 253 specimens, we obtained 214 sequences, and identified 83 operational taxonomic units including leaf‐mining Lepidoptera, Diptera, Coleoptera, and their hymenopteran parasitoids, plus 31 non‐target sequences. Of the 214 sequences, 69 were parasitoid wasps (32.2%). Of these, 40 were obtained from parasitoids dissected directly from leaf mines, while 29 were co‐amplified from leaf‐miner samples, demonstrating that SMRT‐based co‐amplification allows detection of parasitism without rearing. Our analysis revealed 156 trophic interaction types and 247 interaction events. Network analyses showed a highly modular tripartite network, with module structure primarily driven by host plant identity and a reduced set of influential leaf‐miner and parasitoid taxa. Bipartite networks supported the hypothesis that interactions are more strongly compartmentalized between leafminers and host plants than at higher trophic levels. These results demonstrate that long‐read DNA barcoding with co‐amplification detection provides a scalable framework for reconstructing multi‐trophic interactions from individual specimens, overcoming key limitations of rearing‐based methods and enabling robust biodiversity and ecological network assessments in species‐rich ecosystems.

## 1 Introduction

Trophic interactions are the foundation of ecological networks, driving energy flow and nutrient cycling across ecosystems. Among these, interactions between plants, herbivores, and their natural enemies are critical for maintaining biodiversity and ecological stability (Zhang et al. 2018). However, the current biodiversity crisis—marked by species extinction rates 100 to 1000 times higher than historical baselines—is disrupting these networks globally (Pimm et al. 1995; Evans et al. 2013; Díaz et al. 2019). This disruption is especially severe in biodiversity hotspots of the Global South, where conservation infrastructure is often insufficient (Almond et al. 2022). Understanding how host plant associations shape insect herbivore communities and their natural enemies is essential to uncovering the mechanisms that determine community structure and ecosystem function (Janz 2011; Blanchet et al. 2020; Wan et al. 2021).

Tropical dry forests (TDFs) are among the most diverse and yet vulnerable tropical ecosystems, covering about 40% of the world's tropical forest regions and providing key ecosystem services (Quesada et al. 2009). In Mexico, TDFs host a high number of endemic plant and animal species and represent ecologically significant systems, though more than half of their original extent has been lost or degraded due to agriculture, deforestation, cattle ranching, and climate change (Sanchez‐Azofeifa et al. 2013). This degradation not only threatens biodiversity, but also disrupts the trophic interactions essential for ecosystem resilience. Investigating how species interact within these forests is thus critical for understanding the broader ecological consequences of habitat disturbance. Plant–herbivore–parasitoid interactions in tropical dry forests have been poorly studied, despite their potential role in shaping community structure and modulating the effects of environmental change (Cuevas‐Reyes et al. 2007). Addressing this gap is essential to uncover the hidden dynamics that govern biodiversity persistence and ecosystem functioning in these vulnerable biomes.

Leaf‐mining insects offer a powerful model for studying trophic interactions due to their high host specificity (Novotny et al. 2010; Maunsell et al. 2016; Volf et al. 2017; Jorge et al. 2017; Perez‐Hernandez et al. 2022), conspicuous larval feeding traces on leaves that enable efficient detection and sampling in the field (Lopez‐Vaamonde, Kirichenko, and Ohshima 2021; Eiseman 2026) and high susceptibility to parasitoid attack (Lopez‐Vaamonde et al. 2005; Valladares et al. 2001; Cagnolo et al. 2011). Typically monophagous or oligophagous (Chen et al. 2025), these insects form close associations with their host plants, which also mediate interactions with their natural enemies such as parasitoids (Valladares et al. 2001; Lopez‐Vaamonde et al. 2005; Salvo et al. 2011). Their population dynamics are strongly influenced by dominant plant species (Dai et al. 2018) and successional stages, making them sensitive indicators of ecological change (Valladares et al. 2006). These interactions form complex plant–leafminer–parasitoid networks (Memmott et al. 1994; Valladares and Salvo 1999; Valladares et al. 2001; Lewis et al. 2002; Cagnolo et al. 2011; Chen et al. 2025). One key determinant of plant–leafminer–parasitoid network architecture is host specificity and high modularity (Hirao and Murakami 2008; Leppänen et al. 2013; Volf et al. 2017; Chen et al. 2025). However, researchers face challenges in documenting such networks primarily because immature stages of leafminers are sometimes difficult to identify and rearing their associated parasitoids to adulthood is often unsuccessful (Hrcek et al. 2011; Pocock et al. 2012; Leppänen et al. 2013; Lees et al. 2014; Derocles et al. 2015). The use of DNA barcoding as an identification tool can help accurately validate these interactions and detect those that cannot be clarified through the rearing of leafmines (Rougerie et al. 2011; Hrcek et al. 2011; Derocles et al. 2015; Roslin and Majaneva 2016; Hrcek and Godfray 2015; Mutanen et al. 2020; Miller, Aguilera, et al. 2021; Miller, Polaszek, and Evans 2021).

Although DNA barcoding has long been established for species identification of insects in general and leafminers in particular (Kirichenko et al. 2019; Lopez‐Vaamonde, Kirichenko, Cama, et al. 2021), its potential for resolving trophic interactions, particularly in systems with concealed or semi‐concealed hosts (i.e., leafminers, leaf rollers, leaf tiers, case‐bearers, borers, root feeders), remains underutilized (Hrcek et al. 2013). Emerging studies show that DNA barcoding enhances food web resolution (Wirta et al. 2014; Roslin and Majaneva 2016) and enables the detection of hidden or co‐amplified species (such as parasitoids within host tissue) (Traugott et al. 2006, 2008; Rougerie et al. 2011; Derocles et al. 2012, 2015; Kitson et al. 2019; Nakadai and Kawakita 2017; Mutanen et al. 2020; Miller, Aguilera, et al. 2021), and allows for cost‐effective biodiversity monitoring at large scales (Hebert et al. 2025). Metabarcoding has also been successfully used to simultaneously identify multiple interacting insect herbivores and their parasitoids from mixed samples (Sow et al. 2019; Miller, Polaszek, and Evans 2021; Borsato et al. 2025). Nevertheless, the integration of these methods into full trophic network analyses is still in its infancy (Traugott et al. 2013; Hrcek and Godfray 2015; Roslin and Majaneva 2016; Nagarajan et al. 2018; Fang et al. 2024) in particular in tropical ecosystems (Novotny et al. 2010).

In this study, we investigate tri‐trophic interactions among host plants, leaf‐mining insects, and their parasitoids in a Mexican tropical dry forest. We applied Single Molecule, Real‐Time (SMRT) sequencing on single leaf‐mining larvae through the PacBio Sequel workflow at the Canadian Centre for DNA Barcoding (CCDB, Centre for Biodiversity Genomics, University of Guelph) (Hebert et al. 2018), and leveraged co‐amplified sequences—typically discarded—to directly infer host–herbivore–parasitoid linkages. We then used these data to test the hypothesis that leaf‐mining insects are more host‐specific than their associated parasitoids, predicting that networks should be more modular between leafminers and their host plants than between parasitoids and leafminers (Cagnolo et al. 2011). We also identified key taxa driving network structure to evaluate the role of plants as primary bottom‐up drivers of multi‐trophic interactions.

By integrating advanced molecular techniques with morphological identification of host plants and ecological network analysis, this study reveals previously undocumented interactions and provides novel insights into the structure and function of multi‐trophic networks in a tropical forest. Beyond documenting new interactions, this work establishes a powerful new framework for studying insect trophic relationships, with the potential to fundamentally reshape how complex invertebrate interaction networks are investigated. Our findings underscore the value of molecular tools for overcoming taxonomic impediments and advancing biodiversity research, ultimately informing more effective conservation strategies for tropical dry forests.

## 2 Methods

### 2.1 Study Site Description

**FIGURE 1:**
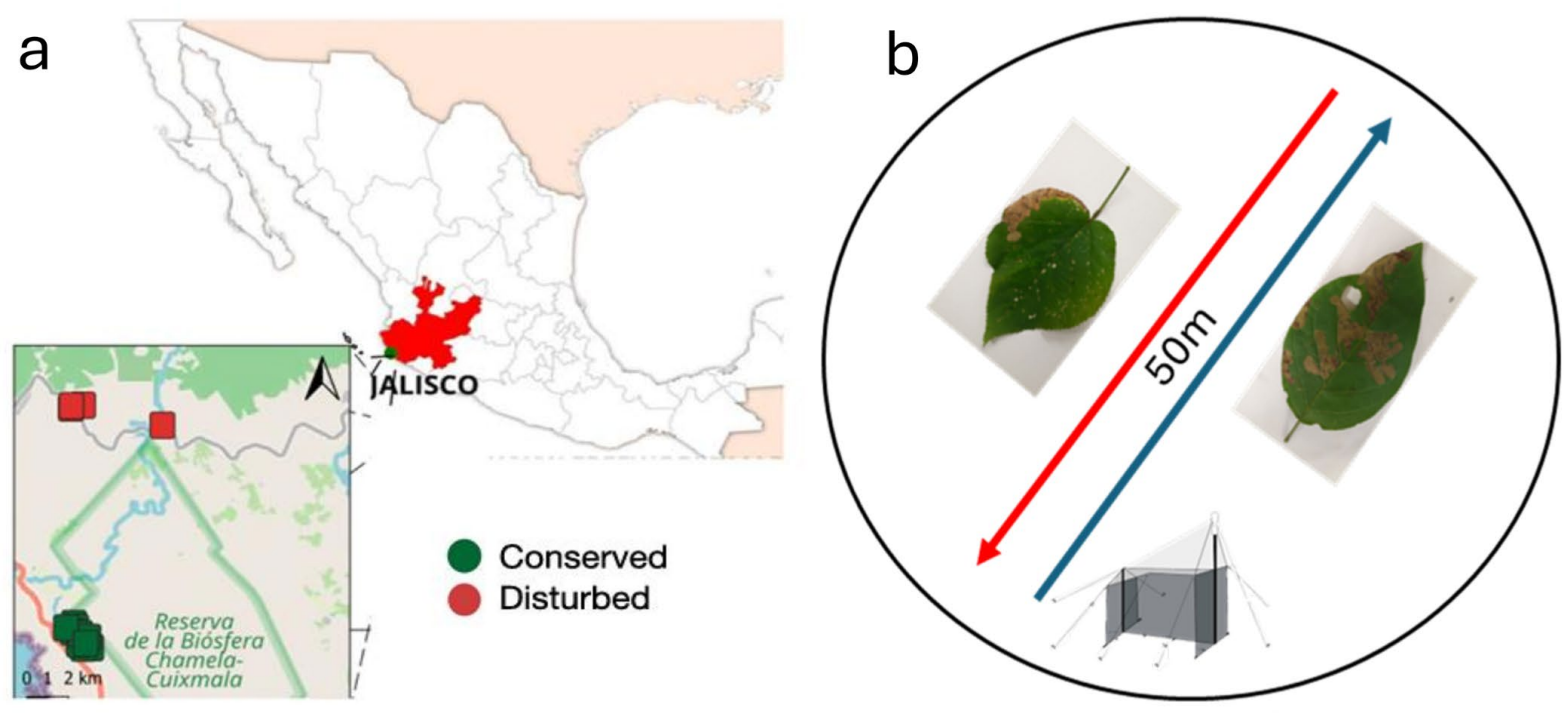
Study area and sampling design in the Chamela‐Cuixmala Biosphere Reserve. Sampling was conducted during the rainy season of 2022, with collections carried out in August, September, and November. The reserve, located in Jalisco, western Mexico, is characterized by tropical dry forest vegetation. Sampling sites encompassed a range of habitat conditions and successional stages. (a) Map of the Chamela‐Cuixmala Biosphere Reserve showing the location of leaf mine collection sites. Green squares indicate sites within the conserved core area, while red squares represent surrounding disturbed locations. (b) Schematic representation of the sampling protocol at each site. Leaf mines were manually collected along a 100‐m linear transect, extending 50 m back and forth from a central point marked by a Malaise trap. The image illustrates mined leaves from both sides of the transect, emphasizing systematic collection across the microhabitat gradient.

This study was conducted at the Estación de Biología Chamela (N 19.49794, W −105.04378), located within the Chamela‐Cuixmala Biosphere Reserve in Jalisco, Mexico (hereafter referred to as Chamela; Figure 1). This site is one of the most plant species‐rich tropical dry forests in the Neotropics, harbouring over 1100 species of vascular plants including 229 species of trees and 227 species of shrubs (Lott 1993; Lott and Atkinson 2006). In the Chamela region, 40% of the flora is distributed on the Pacific slope from Sonora and Baja California Sur to Central America (Lott and Atkinson 2006). The reserve covers over 13,000 ha and includes a mosaic of ecosystems ranging from tropical dry forest to wetlands and coastal vegetation (Noguera et al. 2002). The forest is characterized by strong seasonality, with most trees shedding their leaves during the dry season from October to May. Annual precipitation ranges from 600 to 1200 mm, concentrated in the rainy season between June and September (Takano‐Rojas et al. 2023). This high level of biodiversity and habitat heterogeneity makes Chamela an exceptional natural laboratory for studying ecological interactions (Cuevas‐Reyes et al. 2007) and informing conservation strategies in tropical dry ecosystems.

### 2.2 Taxa Sampling

Leaf mines were manually collected from 10 distinct locations within the core area of the reserve and four surrounding disturbed locations (Figure 1a; Table 1). Sampling was conducted across both conserved and disturbed forest sites to capture a broad range of ecological conditions; however, habitat characteristics were not explicitly incorporated into the analyses, and all network analyses were performed on pooled data because site‐level sample sizes were insufficient for formal comparisons or for disentangling spatial and habitat effects. Collections were conducted during the rainy season of 2022, in August, September, and October. The collection of leaf mines and their dissection was performed following the method described by Lopez‐Vaamonde, Kirichenko, and Ohshima (2021). Briefly, manual collection of leafminers and their host plants was carried out along a 100‐m transect (spanning 50 m back and forth) from the starting point, to capture variation in microhabitats and plant communities (Figure 1b). All occupied mines were dissected under a stereomicroscope at the Chamela Research Station, and any insects (adults, immature stages, or moult remains) found inside the mines were individually transferred to 96‐well plates with 30 ul 96% ethanol in each well for downstream molecular analyses. Plant material from which the mines were dissected was pressed and dried for identification of the host. Some host plants were identified in the field or using the UNAM Open Data Portal (https://datosabiertos.unam.mx/). An effort was made to identify the different plant species at least to the family level. Plants that could not be confidently assigned to a family were grouped into morphospecies based on leaf morphology and, when possible, their associated leafminer. These specimens were labelled as indeterminate (indet).

**TABLE 1:** Sampling sites within the Chamela‐Cuixmala Biosphere Reserve used for the collection of leaf‐mining insects. The table lists the site ID, name, geographic location (latitude and longitude), and estimated canopy cover.

Site	Name	Latitude	Longitude	Canopy coverage (%)
T1	Chachalaca1	19.4969806	−105.04355	67.71
T2	Chachalaca2	19.4958417	−105.041956	48.96
T3	Camino Sur1	19.4959917	−105.039317	77.71
T4	Camino Sur2	19.4971667	−105.041056	83.75
T5	Búho1	19.4990556	−105.041878	86.25
T6	Búho2	19.4988028	−105.040278	79.79
T7	Antiguo norte1	19.5002944	−105.045417	86.46
T8	Antiguo norte2	19.5013889	−105.047483	89.38
T9	Tejón1	19.5014444	−105.043672	91.88
T10	Tejón2	19.5027861	−105.044794	91.46
T11	Calandria	19.504887	−105.037776	*NM*
T12	Baldomero	19.5997972	−105.043406	83.54
T13	Valente Ranchito	19.5922083	−105.009169	91.25
T14	Ranchito Agustin	19.6143028	−105.020889	88.33

### 2.3 Taxa Identification, DNA Barcoding and Coamplifications

Samples were initially identified based on the morphology of the insect specimens found inside dissected leaf mines, as well as on leaf‐mine morphology. In addition, insect specimens were DNA‐barcoded, and the resulting sequences were identified using the Barcode of Life Data Systems (BOLD) ID engine and BIN assignment. Sequences were also clustered into operational taxonomic units (OTUs) using the Multiplex Barcode Research And Visualization Environment (mBRAVE).

Three specimen microplates contained a total of 253 samples: 214 larvae, 29 pupae, six moult remains of leafminers. In addition, we included four adults, three parasitoids that were reared from leaf mines and one weevil that was found alive inside its leaf‐mine (Table S1). These plates were processed as part of a larger CCDB sequencing run that included 93 additional plates from other projects; thus, the laboratory workflow and sequencing reactions described below apply to the full run, of which our samples represent only a small subset.

DNA extraction, PCR amplification and sequencing were carried out at CCDB following their standard high‐throughput DNA barcoding pipeline for arthropods (Ivanova et al. 2006; deWaard et al. 2008; Ratnasingham and Hebert 2007). DNA extraction was performed by a solid phase reversible immobilization (SPRI) method. The 658‐bp barcode fragment of the mitochondrial cytochrome c oxidase subunit I (COI) gene was amplified using CLepFol primer cocktails, with PCR reactions performed in 384‐well format in 6ul reaction volumes using 1 μL of DNA extract per reaction. Each DNA extract was amplified in duplicate, yielding a total of 9216 unique PCR reactions. Because our samples represented 506 of the unique PCR reactions in the full sequencing run (≈5.5%), they are expected to account for a similar proportion of the total CCS reads generated. Single‐step PCR with fusion primers produced asymmetrically labelled dual‐indexed amplicons suitable for multiplexing, a strategy that improves sequencing efficiency while minimizing index hopping and barcode crosstalk (Kozich et al. 2013). Amplification products were pooled, quantified, and sequenced on a PacBio Sequel IIe platform using a single 8 M SMRT Cell. Library preparation followed the Express Template Prep Kit v2.0, with sequencing performed using the Sequel Binding Kit v2.1 and Sequencing Reagent Plate v2.0, and data collected using a 10‐h movie under circular consensus sequencing conditions optimized for high‐fidelity long reads (Wenger et al. 2019). Sequence assembly, quality control, and taxonomic assignment were conducted within BOLD and mBRAVE workflows.

Circular Consensus Sequencing (CCS) reads were generated using PacBio's CCS algorithm, applying a minimum predicted accuracy threshold of 99.9%. Reads shorter than 500 base pairs or longer than 1000 base pairs were excluded to ensure data quality, yielding a total of 2,213,540 high‐confidence CCS reads.

These reads, along with indexed primer sequences and sample metadata, were uploaded to mBRAVE, a cloud‐based platform designed for large‐scale metabarcoding analysis and ecological inference (Ratnasingham 2019). Demultiplexing was conducted using perfect index matching, followed by rigorous quality filtering, OTU clustering, and taxonomic assignment. During preprocessing, 50 bases were trimmed from both ends of each read, and only sequences with a minimum Phred quality score of 50 were retained. Additional quality thresholds excluded reads with more than 1.0% of bases below QV 20% and 0.5% below QV 10. OTU formation used a 2% sequence identity threshold and required a minimum overlap of 100 base pairs, with outlier sequences exceeding a 2.5% mismatch excluded from clustering. Taxonomic assignments relied on default Barcode Index Number (BIN) reference libraries provided by BOLD Systems.

To distinguish primary from secondary sequences within each DNA extract, assembled contigs were categorized as dominant or non‐dominant based on their relative abundance within each sample. Dominant contigs, defined as the most abundant clusters recovered from each sample (i.e., assembled from the highest read counts), typically corresponded to the focal organism, in this study, the leaf‐mining insect, with read counts ranging from 4 to 124. Non‐dominant contigs, with read counts ranging from 1 to 34, often represented co‐amplified sequences such as parasitoids, microbial contaminants, or environmental DNA. This classification enabled the inference of multi‐trophic interactions while minimizing the risk of false positives due to background noise or laboratory artefacts. Only samples in which dominant contigs matched known or expected insect herbivores and non‐dominant sequences passed contamination filters (e.g., non‐bacterial, non‐chimeric) were included in ecological network analyses. Retained non‐dominant sequences were assigned the same sample ID as their respective hosts, with the addition of a “.NTS1” (non‐target sequence) suffix; in two cases where two subdominant sequences were retained per host, an additional suffix “.NTS2” was used (Tables 1 and 2).

**TABLE 2:** Observed network metrics and standardized effect sizes (Z‐scores) for bipartite and tripartite interaction networks involving host plants, leafminers, and parasitoids. Metrics include Nestedness (NODF2), Connectance, Modularity (Q), and network‐level specialization (H2′). Z‐scores were calculated by comparing observed values against null distributions generated from 1000 marginal‐preserving random networks using the Patefield algorithm, as implemented in the r2dtable (r2d) function. Positive Z‐scores indicate higher‐than‐expected values relative to random expectations, whereas negative Z‐scores indicate lower‐than‐expected values.

Network	Metric	Observed	Z_score	Significance
Leafminer‐Plant	Nestedness	7.77	2.75	0.008 (*p* < 0.01**)
	Connectance	0.03	−19.32	1.000
	H2′	0.84	25.71	0.002 (*p* < 0.01**)
	Modularity (Q)	0.88	15.05	0.002 (*p* < 0.01**)
Parasitoid‐Leafminer	Nestedness	11.02	−0.37	0.590
	Connectance	0.06	−9.89	1.000
	H2′	0.53	10.93	0.002 (*p* < 0.01**)
	Modularity (Q)	0.79	8.35	0.002 (*p* < 0.01**)
Parasitoid–Plant	Nestedness	8.91	0.16	0.163
	Connectance	0.06	−7.07	1.000
	H2′	0.49	7.67	0.002 (*p* < 0.01**)
	Modularity (Q)	0.73	5.09	0.002 (*p* < 0.01**)
Tripartite	Modularity (Q)	0.80	30.45	0.001 (*p* < 0.001***)

*Note:* Significance levels are indicated as follows: *p* < 0.01 (**), *p* < 0.001 (***).

A neighbour‐joining (NJ) tree was generated in BOLD (Figure S1).

### 2.4 Analysis of Trophic Interactions

To construct and compare interaction networks among leafminers, host plants, and parasitoids, we used the Bipartite package (version 2.18) within R (version 4.3.1). Data from August, September, and October were pooled to represent the entire sampling season. The bipartite package offers a suite of tools for visualizing bipartite ecological networks and computing various network‐level indices, facilitating the analysis of complex trophic relationships (Dormann et al. 2009). When parasitoids were detected solely through BIN identification without an associated leafminer BIN from the same sampling event, interaction links were established using morphological identification of the leaf mine and its associated host plant, when possible. This approach allowed us to retain ecologically plausible interactions even in the absence of full molecular triads.

This analysis incorporated Barcode Index Numbers (BINs) assigned to insect DNA sequences, morphologically identified host plants, and taxonomically resolved co‐amplified sequences. Each BIN or co‐amplification, whether corresponding to a leafminer or parasitoid, was treated as a molecular operational taxonomic unit (MOTU) and represented as a node within the interaction network. For host plants, all identified taxa—regardless of resolution to species, genus, or morpho‐species—were included as distinct nodes. To quantify network structure, we applied a suite of metrics designed to capture patterns of specialization, modularity, and interaction asymmetry.

First, we estimated nestedness using NODF2 (Nestedness based on Overlap and Decreasing Fill; Almeida‐Neto et al. 2008; Ulrich et al. 2009). This metric quantifies the extent to which specialist species interact with a subset of the partners used by more generalist species, rather than forming entirely unique interactions. A network with a nestedness value of 0 indicates no nested structure, while a value of 100 represents perfect nestedness. Then we estimated connectance, a metric that divides the observed number of interactions by the total number of possible interactions (Jordano 1987). To test whether groups of species within the networks interact more strongly among themselves, we performed a Q modularity test for weighted bipartite networks with 100 independent runs using the null model‐generated networks (r2d), generating only one modular configuration for each network (Dormann and Strauss 2014). Finally, we estimated the specialization index H2′ that indicates increasing specialization within modules with values from 0 to 1 (Blüthgen et al. 2006). To determine whether network metrics are the result of random interactions, we compared each network metric with the null model suggested by Dormann et al. (2009), which keeps the matrix and row/column sums constant. We used *N* = 1000 permutations with the r2dtable null model (Patefield model, 1981) function in the ‘bipartite’ R package (Dormann et al. 2008).

### 2.5 Network Analyses

To investigate the structure and organization of the ecological networks, we reconstructed both bipartite and tripartite interaction graphs and quantified their properties using complementary graph‐theoretical and machine‐learning approaches.

### 2.6 Network Reconstruction

Bipartite interaction networks were reconstructed using the igraph package v2.0.1 (Csardi and Nepusz 2006). Quantitative interaction matrices were converted into biadjacency graphs, with nodes assigned to ecological layers following standard bipartite conventions (Dormann et al. 2009). Module identities obtained from the QuanBiMo algorithm were appended as node attributes, enabling subsequent analyses of network topology, interaction strength, and community structure within a unified graph framework.

A tripartite interaction network linking host plants, leafminers, and parasitoids was constructed by integrating the two bipartite interaction matrices into a unified edge list, in which each interaction retained its trophic identity (plant–leafminer or leafminer–parasitoid). The graph was generated using graph_from_data_frame() in igraph (Csardi and Nepusz 2006), assigning each node to one of three trophic categories (“Plant”, “Leaf_Miner”, or “Parasitoid”). Isolated nodes were removed prior to analysis to avoid artificial inflation of network statistics, and network visualization was performed using ggraph (Pedersen 2025).

### 2.7 Network Metrics and Modularity

For bipartite networks, network‐level properties were quantified using the bipartite package (Dormann et al. 2008), including connectance, nestedness (NODF; Almeida‐Neto et al. 2008), specialization (H2′; Blüthgen et al. 2006), and modularity (Q). For the tripartite network, only modularity was evaluated because connectance, nestedness, and specialization are formally defined for bipartite systems and are not directly comparable in multilayer ecological networks (Kéfi et al. 2016; Pilosof et al. 2017).

Community structure in the tripartite network was inferred using the Louvain algorithm, a fast and widely used modularity‐maximization method (Blondel et al. 2008). To reduce stochastic variation, the optimization was repeated 100 times using different random seeds, and the partition with the highest modularity (Q) was retained for downstream analyses.

### 2.8 Null‐Model Analyses

To evaluate whether observed network properties differed from random expectations, we generated 1000 null networks using the marginal‐preserving r2d algorithm (Bascompte et al. 2003). For each null network, the corresponding network metrics were calculated and summarized by their mean, standard deviation, Z‐score, and one‐tailed probability (P_null ≥ observed). Observed values were compared with these null distributions to assess whether network structure departed from random expectations.

### 2.9 Random Forest Classification of Network Modules

To identify the taxa contributing most strongly to network modularity, we applied Random Forest classification separately to the bipartite and tripartite networks using the randomForest package (Breiman 2001; Liaw and Wiener 2002). Weighted adjacency matrices were converted into supervised datasets in which taxa represented observations, interaction strengths served as predictor variables, and module identity was the categorical response. A forest of 500 trees was used to calculate Mean Decrease Gini (MDG), an impurity‐based measure of variable importance widely used in ecological multivariate analyses (Cutler et al. 2007). Taxa with the highest MDG values were retained for subsequent multivariate analyses.

### 2.10 Gini‐Weighted PCA of Modular Structure

To visualize multivariate patterns among the taxa contributing most strongly to module identity, we performed principal component analysis (PCA) on centered and scaled interaction profiles using the *prcomp()* function (Jolliffe and Cadima 2016). Taxon loadings were weighted according to their MDG values, allowing vector lengths to reflect both their contribution to the principal components and their importance in predicting module identity. The resulting Gini‐weighted biplots provide a low‐dimensional representation of network organization and highlight the taxa that most strongly shape modular structure.

### 2.11 Genetic Distances and Interaction Modules

To assess whether taxonomically related species exhibit similar interaction patterns, we quantified pairwise genetic distances among leafminers and parasitoids directly from DNA barcode sequences, using the K80 evolutionary model implemented in Ape version 5.0 in R (Paradis and Schliep 2019). The K80 model was selected as it provided the best fit to the data according to the corrected AIC criterion in jModelTest (Posada 2008). These distances were calculated directly from sequence divergence and do not rely on an explicit phylogenetic reconstruction; they are therefore interpreted as proxies for taxonomic relatedness among taxa rather than fully resolved phylogenetic relationships. Distances were calculated separately for each of the three ecological networks, retaining only the upper trophic levels (leafminers and parasitoids) and excluding host plants. We addressed this question using two complementary approaches.

First, we evaluated whether taxonomically related species tend to share similar interaction partners by performing Mantel tests between genetic distance matrices (K80) and ecological distance matrices. Ecological distances were calculated using the Jaccard dissimilarity index on binary interaction matrices, implemented in the R package vegan (Oksanen et al. 2019). Mantel tests were conducted using Pearson correlation with 9999 permutations.

Second, we assessed the genetic coherence of interaction modules using a permutation‐based approach. For each network, we calculated the observed mean pairwise genetic distance (K80) within modules and between modules, and compared these values against a null distribution generated from 9999 permutations, constrained to preserve the original size of each module, following Cagnolo et al. (2011). Modules containing only one BIN were excluded from within‐module calculations. From this null distribution, we derived Z‐scores and associated *p*‐values for both within‐ and between‐module comparisons.

## 3 Results

### 3.1 Insect Species Richness

**FIGURE 2:**
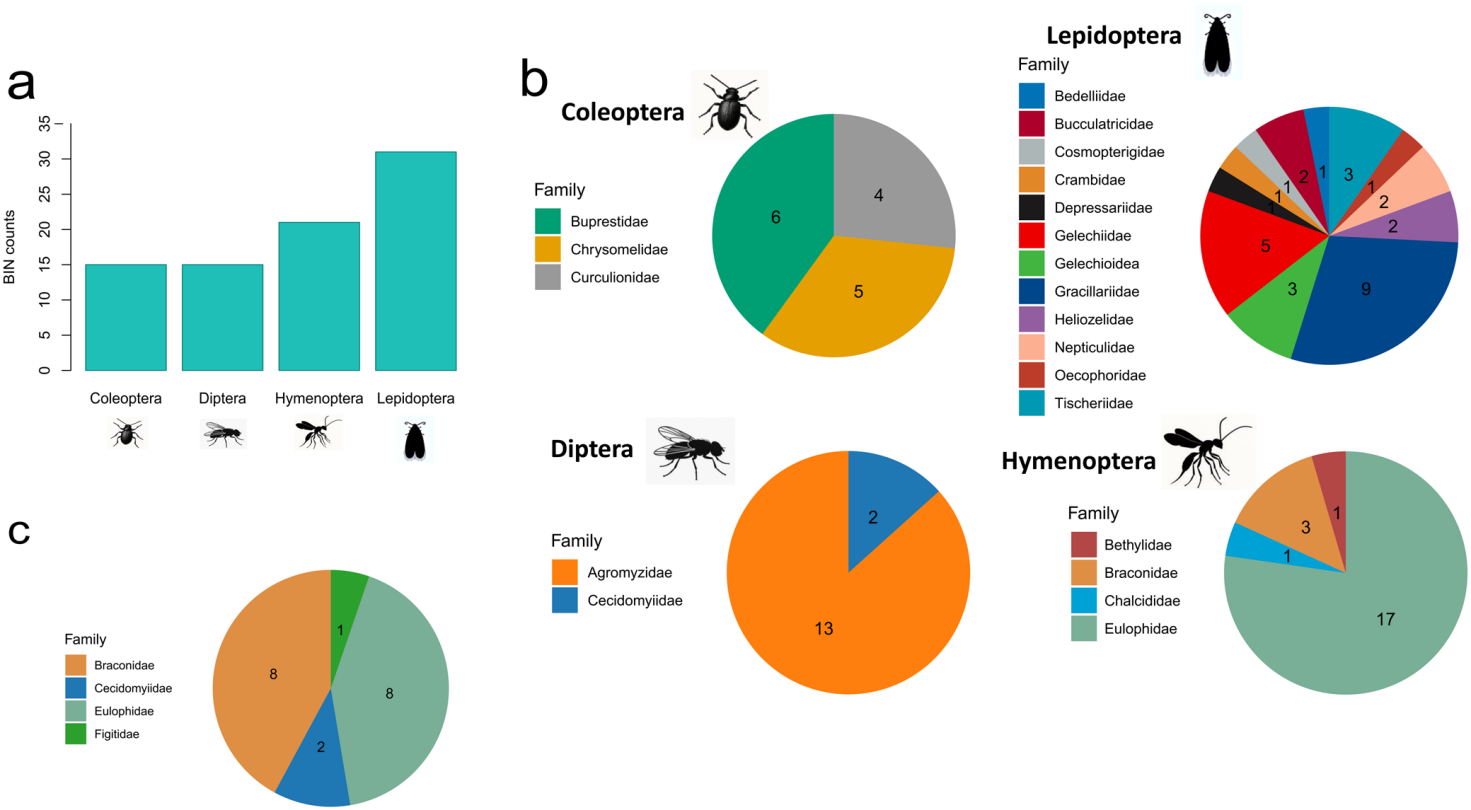
Taxonomic composition of leaf‐mining insects and associated parasitoids recovered by DNA barcoding. (a) Number of Barcode Index Numbers (BINs) recovered for each insect order obtained from dominant contigs. (b) Family‐level composition within each order. Pie charts show the number of BINs per family for Coleoptera, Diptera, and Lepidoptera. Numbers within pie slices indicate BIN counts per family. (c) Number of OTUs of Co‐amplified non‐target sequences per insect family.

Of the 253 specimens collected from 161 tenanted leaf mines, 214 yielded sequences (84.6% success rate), of which 212 were assigned to 81 distinct BINs. Two additional sequences contained stop codons but were nonetheless identified and included, resulting in a total of 83 OTUs. Out of the 161 leaf mines collected, 27 contained more than one leaf‐mining larva inside (22 Lepidoptera leaf mines, four Coleoptera, and one Diptera). Parasitoids were obtained from 35 leaf mines, and only six of these yielded more than one parasitoid per mine (Table S2). The taxonomic breakdown revealed 31 OTUs in Lepidoptera, 21 in Hymenoptera, and 15 each in Diptera and Coleoptera (Figure 2a). Across these groups, 21 families were represented: 12 lepidopteran, four hymenopteran, three coleopteran, and two dipteran (Figure 2b, Table S1).

Among Lepidoptera, the most species‐rich family was Gracillariidae (9 BINs), followed by Gelechiidae (5), and Tischeriidae (3 each), and Heliozelidae, Nepticulidae, and Bucculatricidae (2 each). We found three BINs that could only be assigned to the superfamily Gelechioidea. Single BINs were identified for Cosmopterigidae, Depressariidae, Oecophoridae, Bedelliidae, and Crambidae. For Coleoptera, Buprestidae was the most species‐rich family (6 BINs), followed by Chrysomelidae (5) and Curculionidae (4). In Diptera, nearly all BINs belonged to Agromyzidae (13 BINs), while two Cecidomyiidae BINs that were excluded from core analyses due to uncertain leafminer status were retained as co‐amplifications.

For Hymenoptera, all 21 BINs corresponded to families with known parasitoids (including hyperparasitoid) or parasitoid‐associated life histories. Eulophidae was the most represented family (17 BINs), followed by Braconidae (3), with Chalcididae and Bethylidae each represented by one BIN. Only 17 BINs could be assigned to genus level (representing 13 genera), and 4 BINs were identified at species level (corresponding to three species; Table S1).

Of the 81 Barcode Index Numbers (BINs) recovered, 48 (57.8%) are new to the BOLD database (Table S2). Novelty varied by taxonomic group: Coleoptera showed the highest proportion of new BINs (93.3%), followed by Hymenoptera (63.6%), Lepidoptera (45.2%), and Diptera (40%). Most previously known Dipteran BINs belong to Agromyzidae, reflecting their better historical sampling coverage.

Among the 35 previously recorded BINs, 21 had been documented in Mexico, all in Chamela, while 22 were also found in Costa Rica's Guanacaste tropical dry forest. Additional shared BINs were found with Panama (2 BINs) and Canada (1 BIN), indicating wider Neotropical–Nearctic connectivity. Four BINs were identified to species level and exhibited continental distributions, including *Calycomyza malvae* , *Closterocerus utahensis*, and *Paratischeria neotropicana*, spanning from North to South America (Table S2).

### 3.2 Analysis of Non‐Target Sequences

Out of 243 leaf‐mining immature stages screened (214 larvae and 29 pupae), we recovered 31 non‐target sequences (NTS) from subdominant contigs, representing 12.8% of samples. These NTS corresponded to co‐amplified parasitoid DNA, enabling the detection of parasitism events directly from single leaf‐mining hosts without the need for rearing. Nineteen of these were assigned to 12 different BINs, allowing the identification of seven OTUs (Table S2). Six of the 31 NTS contained stop codons and were therefore excluded from BIN assignment (Table S1). As with the two pseudogenes obtained from dominant contigs, these sequences were nonetheless identified using the BOLD ID engine and were included in the analysis as OTUs (Table S2).

In total, we obtained 69 parasitoid sequences from 214 samples, corresponding to a parasitism rate of 32.2%. Of these, 40 sequences were obtained from samples dissected directly from leaf mines, while 29 were co‐amplified from leaf‐mining insects (all from larvae except for one obtained from a dipteran pupa; Tables S1–, S3).

A total of 29 Hymenoptera NTS were obtained. Of these, 17 belonged to the family Braconidae, predominantly to the genus *Mirax* (Tables S1–, S3). The family Eulophidae accounted for 10 co‐amplified sequences, while two additional co‐amplifications belonged to the family Figitidae. The remaining two NTS were Diptera, both assigned to the family Cecidomyiidae (Tables S1–, S3). These dipteran co‐amplifications were retained in the analysis because they likely represent genuine predatory interactions with leafminers, as previously reported in the literature (Eiseman 2026).

We detected two cases of possible hyperparasitism, each involving two NTS obtained from a single leaf‐mining larva (Table S3). In the first case, one eulophid (BOLD:ADQ2174) and one braconid (BOLD:AFK1014) were recovered from a larva of the agromyzid *Calycomyza malvae* (BOLD:ADT0837). In the second case, the same eulophid species (BOLD:ADQ2174) and a Figitidae (OTU418) were sequenced from another larva of *Calycomyza malvae* .

### 3.3 Assessing Insect Host‐Plant Range

Leaf‐mining insects and their hymenopteran parasitoids were associated with at least 14 host plant families (Table S2 “Host Plants” column). The most represented families were Fabaceae (55 host individuals), Malvaceae (46), and Euphorbiaceae (13). Other notable families included Sapindaceae (19), Nyctaginaceae (14), and Rhamnaceae (10). The remaining families—Convolvulaceae, Commelinaceae, Asteraceae, Rubiaceae, Bignoniaceae, Cactaceae, Meliaceae, and Salicaceae—each contributed fewer than five individuals.

At the genus level, 19 genera were identified. Fabaceae showed the highest generic diversity, including *Nissolia* (16), *Lonchocarpus* (13), *Rynchosia* (7), *Apoplanesia* (5), *Coursetia* (2), *Coulteria* (3), and *Croton* (1). Other well‐represented genera included *Ayenia* (40) in Malvaceae, *Acalypha* (10) and *Dalechampia* (2) in Euphorbiaceae, *Cardiospermum* (15) in Sapindaceae, *Guapira* (14) in Nyctaginaceae, and *Gouania* (9) in Rhamnaceae (Table S2).

Fifteen plant species were identified, including *Ayenia micrantha* (24), *Cardiospermum halicacabum* (15), *Guapira macrocarpa* (14), *Nissolia leiogyne* (8), *Lonchocarpus eriocarinalis* (8), *Acalypha langiana* (9), *Nissolia fruticosa* (7), *Rynchosia phaseoloides* (6), *Apoplanesia paniculata* (5), and several others with fewer occurrences (Table S2). The taxonomic assignment of the remaining plants could not be determined with certainty, and they were therefore grouped into 18 morphospecies based on leaf or mine morphology, and labelled from Indet 1 to Indet 18. The most abundant of these plant morphospecies was Indet 1, which was associated with nine leafminer specimens.

### 3.4 Trophic Interaction Networks

**FIGURE 3:**
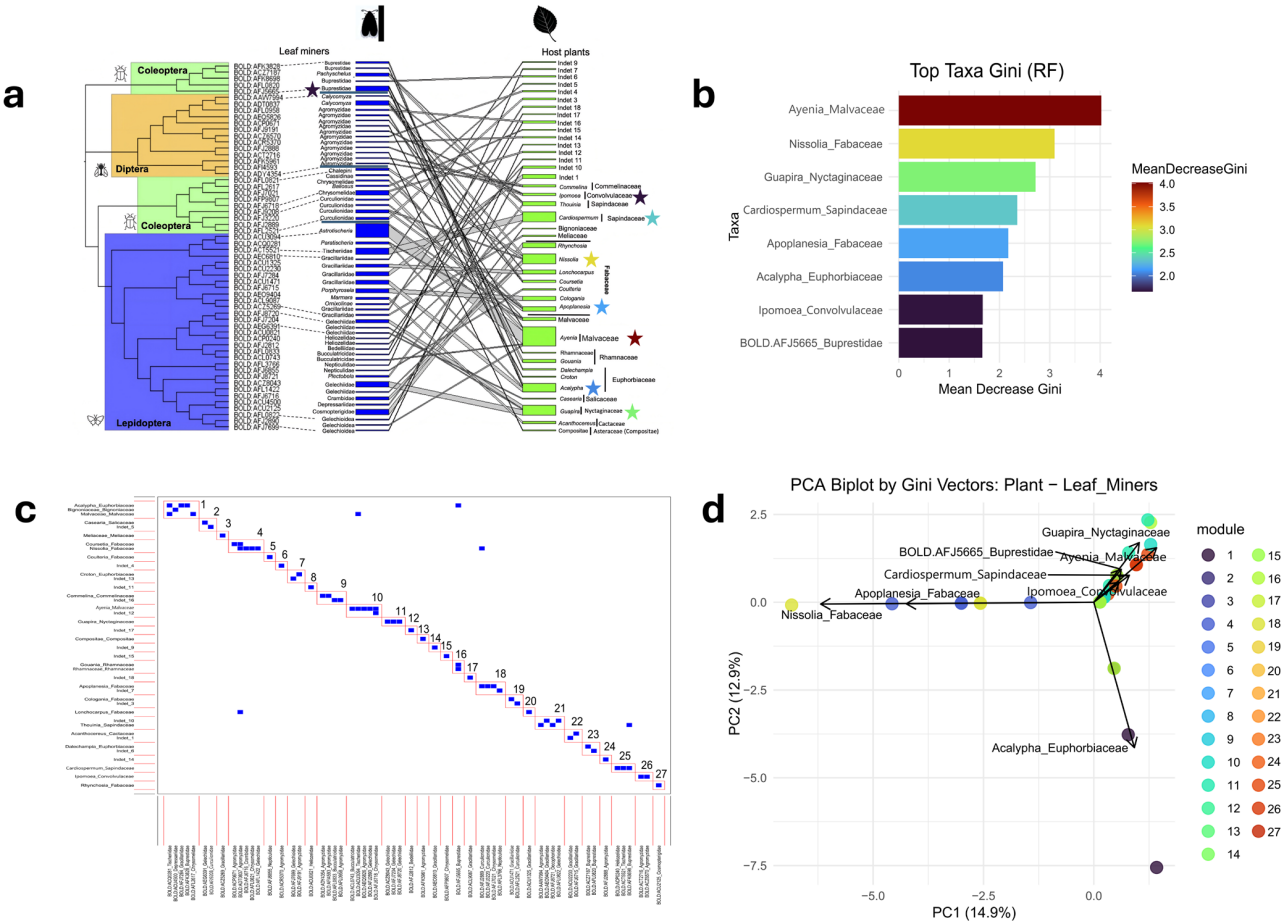
Structure and modular organization of the leafminer–host plant interaction network. (a) Bipartite network linking leafminer BINs (left) to host plant taxa (right). Leafminers are grouped by insect order (Coleoptera, Diptera, Lepidoptera; colour‐coded blocks), and host plants are shown at the family or morphospecies level (Indet). Edges represent observed interactions. Coloured stars highlight host plant taxa identified as influential by Random Forest analyses. (b) Variable importance of the most influential taxa shaping network modular structure, estimated as Mean Decrease Gini from Random Forest classification of modules. Colours indicate relative importance values. (c) Module composition and interaction structure inferred from the QuanBiMo algorithm, showing the assignment of taxa to modules and the distribution of interaction strengths across modules (module numbers indicated). (d) Gini‐weighted PCA biplot of the most influential taxa. Points represent taxa coloured by module membership, and arrows indicate taxa with the highest Gini importance, illustrating gradients of interaction influence structuring the plant–leafminer network.

**FIGURE 4:**
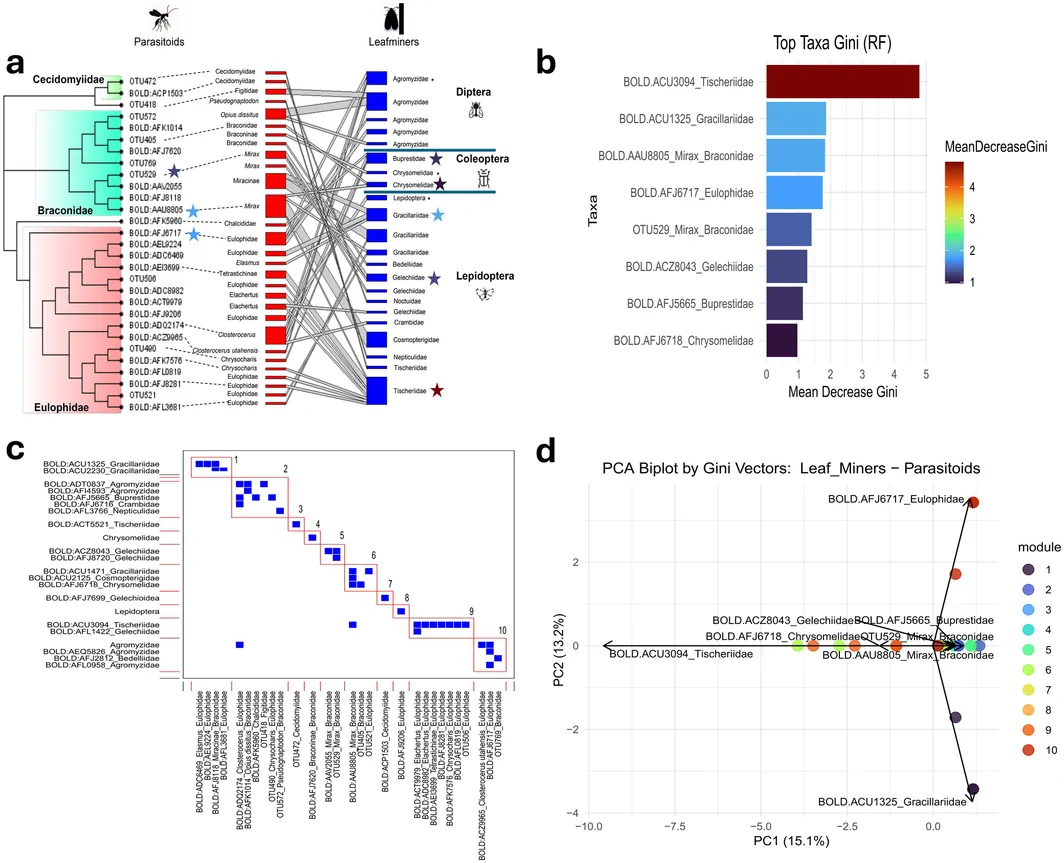
Structure and modular organization of the parasitoid–leafminer interaction network. (a) Bipartite network linking parasitoid BINs (left) to leafminer BINs (right). Parasitoids are grouped by family (Cecidomyiidae, Braconidae, Eulophidae; shaded backgrounds), and leafminers are grouped by insect order (Diptera, Coleoptera, Lepidoptera). Edges represent detected parasitoid–host associations inferred from co‐amplified DNA. Coloured stars indicate taxa identified as influential in Random Forest analyses. (b) Random Forest variable importance (Mean Decrease Gini) for the most influential taxa contributing to module assignment in the parasitoid–leafminer network. Higher values indicate stronger contribution to network structure. (c) Module structure inferred using the QuanBiMo algorithm, showing the distribution of interactions across modules (module numbers indicated by red boxes) and highlighting the strongly modular organization of the network. (d) Gini‐weighted PCA biplot of influential taxa in the parasitoid–leafminer network. Points represent taxa coloured by module membership, and vectors indicate taxa with the highest Gini importance, illustrating gradients of interaction influence structuring parasitoid–host associations.

**FIGURE 5:**
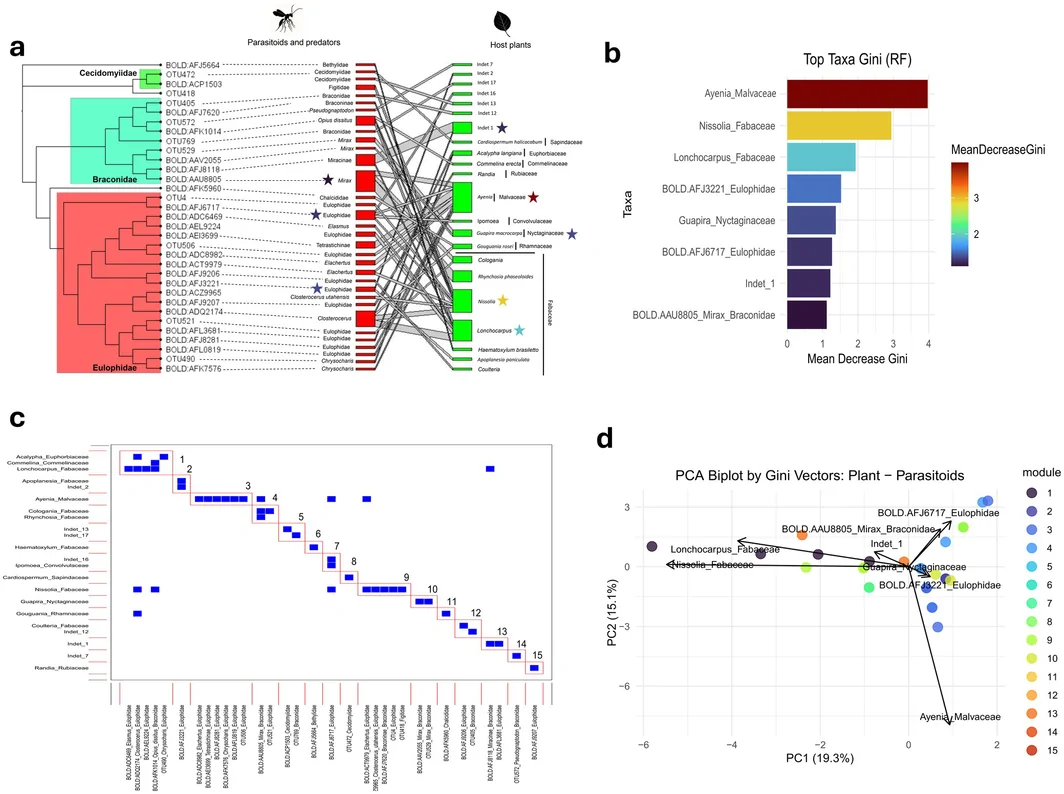
Structure and modular organization of the parasitoid–host plant interaction network (indirect bipartite projection). (a) Bipartite network linking parasitoids and predators (left) to host plants (right), with interactions mediated indirectly by shared leafminer hosts. Parasitoids are grouped by family (Cecidomyiidae, Braconidae, Eulophidae; shaded backgrounds), and host plants are shown as individual taxa (green bars). Edges represent inferred indirect associations derived from the tri‐trophic dataset. Coloured stars highlight taxa identified as influential in Random Forest analyses. (b) Random Forest variable importance (Mean Decrease Gini) for the most influential taxa shaping module assignment in the parasitoid–plant network. Higher values indicate stronger contributions to network structure. (c) Modular structure inferred using the QuanBiMo algorithm, showing the distribution of indirect interactions across modules (module numbers indicated by red boxes), emphasizing the highly compartmentalized organization of the network. (d) Gini‐weighted PCA biplot of influential taxa in the parasitoid–plant network. Points represent taxa coloured by module membership, and vectors indicate taxa with the highest Gini importance, illustrating gradients of influence that structure indirect parasitoid–plant associations.

**FIGURE 6:**
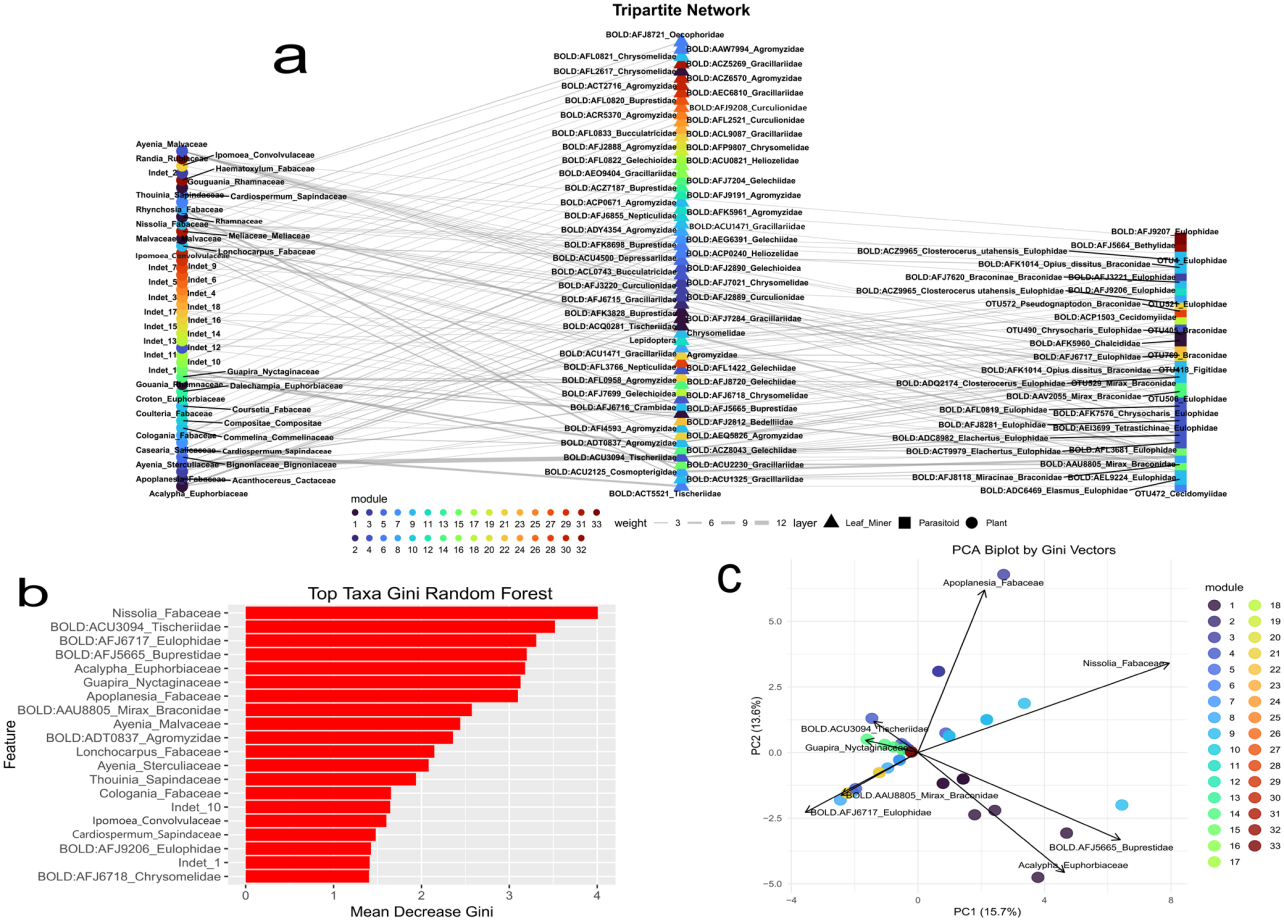
Structure and modular organization of the tripartite interaction network. (a) Tripartite network linking host plants (left), leafminers (center), and parasitoids (right). Nodes are coloured by module assignment as identified by modularity optimization, and edges represent inferred trophic interactions. The network highlights strong compartmentalization into discrete multi‐trophic modules structured primarily by host plants. (b) Top taxa contributing to modular structure based on Random Forest classification, shown as Mean Decrease Gini values. The highest‐ranking features are dominated by plant taxa, followed by key leafminer and parasitoid BINs, indicating that plant identity is the main driver of module differentiation in the tripartite network. (c) PCA biplot of Gini‐weighted interaction profiles for the most influential taxa. Points are coloured by module membership, and vectors indicate the direction and strength of taxa contributions to principal components. The ordination reveals gradients in interaction influence driven mainly by host plants (e.g., *Nissolia* and *Apoplanesia*), with secondary contributions from specific leafminers and parasitoids.

We analysed interaction networks at two levels of complexity: (i) three bipartite networks representing directly (plant–leafminer, parasitoid–leafminer) and indirectly (parasitoid–plant) inferred interactions (Figures 3, 4, 5), and (ii) a tripartite network integrating all three trophic levels (host plants–leafminers–parasitoids; Figure 6).

### 3.5 Leafminer–Host Plant Network

This network comprised 40 host plant taxa (including 16 unidentified morphospecies) and 58 leaf‐miner BINs, connected by 67 interaction types (118 interaction events; Figure 3a; Table S5). Metric values are summarized in Table 2. This network exhibited significantly high modularity and specialization relative to null model expectations (modularity *Q* = 0.88, *z* = 15.05; H2′ = 0.84, *z* = 25.71). Connectance was low (*C* = 0.029) and markedly lower than expected under the null model (*z* = −19.32), although this deviation was not statistically significant. Nestedness was moderately higher than expected by chance (NODF = 7.77, *z* = 2.75), but substantially weaker than the observed modular structure.

MDG values (Figure 3b) indicated that seven plant genera were the most influential nodes shaping network structure. *Ayenia* (Malvaceae) emerged as the most influential taxon (MDG = 4.023), interacting with five leafminer BINs spanning all three insect orders. The most frequent association involved the micro‐moth *Atrotischeria* (BOLD:ACU3094) and species of *Ayenia*, with 12 recorded interactions, forming one of the dominant species‐specific modules. *Nissolia* (Fabaceae) (MDG = 3.094) interacted with a similar number of leafminer BINs but exhibited fewer total interactions, whereas *Guapira* (Nyctaginaceae) (MDG = 2.716) interacted exclusively with three phylogenetically related Gelechiidae BINs. Other influential plant taxa, including *Apoplanesia* (Fabaceae), *Acalypha* (Euphorbiaceae), and *Ipomoea* (Convolvulaceae), served as hosts plants for leafminers belonging to one or two insect orders.

Twenty‐nine of the 31 leafminer BINs (93.5%) were recorded on a single host plant species, family or morphospecies (Figure 3a). The only exception was the lepidopteran *Paratischeria neotropicana* (Tischeriidae, BOLD:ACQ0281), which was associated with both Malvaceae and Euphorbiaceae. The other generalist leafminer is a coleopteran of the family Buprestidae (BOLD:AFJ5665), with interactions with plants in Rhamnaceae and Euphorbiaceae families. This taxon ranked eighth in importance according to the MDG analysis (Figure 3b). Host plant taxonomy was identified at least to the family level for 48 leafminer BINs. Leafminers associated with unidentified host plants (morphospecies labelled Indet) primarily belong to Lepidoptera, followed by Coleoptera and Diptera, highlighting unresolved interactions in the dataset.

We found 27 modules in the leafminer‐host plant network (Figure 3c). According to PCA Biplot using Gini vectors (Figure 3d), plant genera located toward negative values of PC1, such as *Nissolia* and *Apoplanesia* (Fabaceae), are associated with more restricted interaction patterns, whereas genera positioned closer to zero or positive PC1 values, including *Ayenia* (Malaceae), *Cardiospermum* (Sapindaceae), *Guapira* (Nyctaginaceae), and *Ipomoea* (Convolvulaceae). PC2 captured a secondary gradient contrasting modules dominated by *Acalypha* (Euphorbiaceae). Some leafminers provide structure to the network, particularly BOLD:AFJ5665 (Coleoptera: Buprestidae).

### 3.6 Parasitoid–Leafminer Network

This network comprised 22 leaf‐miner BINs and 29 parasitoid BINs, connected by 41 interaction types (64 interaction events; Figure 4a; Table S6). Network metrics (Table 2) show that specialization was significantly higher than expected under the null model (H2′ = 0.535, *z* = 10.93, *p* < 0.01). Modularity was also high (*Q* = 0.794) and significantly greater than null expectations (*z* = 8.35, *p* < 0.01). In contrast, nestedness did not differ from random expectations (NODF = 11.02, *z* = −0.37). Connectance was lower than expected under the null model (*C* = 0.064), but it was not significantly different (*z* = −9.89). The most important parasitoid families were Eulophidae (22 interactions, 53.7%, 32 interaction events) and Braconidae (15 interactions, 36.6%, 27 interaction events). Host ranges in both families included three insect orders (Eulophidae: Lepidoptera 14 BINs 63.6%, Diptera 6 BINs 27.3%, Coleoptera 2 BINs 9.1%; Braconidae: Lepidoptera 10 BINs 66.7%, Diptera 2 BINs 13.3%, Coleoptera 3 BINs 20%).

MDG values (Figure 4b) show that the tischeriid leafminer BOLD:ACU3094 (Lepidoptera: Tischeriidae) exerted a disproportionate influence on network structure (MDG = 4.78). This lepidopteran species showed strong host fidelity with six eulophid parasitoids, as well as associations with the oligophagous eulophid *Elachertus* BOLD:ACT9979 and the more generalist braconid, *Mirax* BOLD:AAU8805. The next most influential taxa included four leafminers and three parasitoids (Figure 4b). Among leafminers, these were the micromoths BOLD:ACU1325 (Lepidoptera: Gracillariidae) and micro BOLD:ACZ8043 (Lepidoptera: Gelechiidae), the jewel beetle BOLD:AFJ5665 (Coleoptera: Buprestidae), and the leaf beetle BOLD:AFJ6718 (Coleoptera: Chrysomelidae). Among parasitoids, the braconid *Mirax* BOLD:AAU8805 was the only braconid recorded parasitizing hosts from multiple leafminer families of different orders Lepidoptera (Tischeriidae, Gracillariidae and Cosmopterigidae) and Coleoptera, (Chrysomelidae). Other parasitoids with strong influence on network topology included BOLD:AFJ6717 (Eulophidae), associated with dipteran leafminers, and OTU529 (*Mirax*, Braconidae), primarily linked to gelechiid hosts. The BOLD:ADQ2174 (*Closterocerus*, Eulophidae) parasitized leafminers from Diptera, Coleoptera, and Lepidoptera, indicating broad host use.

The network was partitioned into 10 modules (Figure 4c). A PCA biplot based on Gini importance vectors (Figure 4d) showed that PC1 was primarily driven by the lepidopteran *Astrotischeria* BOLD:ACU3094, separating modules dominated by specific lepidopteran hosts from those with broader parasitoid–leafminer associations. PC2 was strongly shaped by the eulophid parasitoid BOLD:AFJ6717, while modules centered on the gracillariid leafminer BOLD:ACU1325 occupied the opposite end of this gradient.

### 3.7 Parasitoid–Host Plant Interaction Network

We included the bipartite parasitoid–plant network as an indirect projection of the tripartite system, capturing host‐mediated associations via shared leafminer hosts and complementing the multi‐trophic analysis. This network, representing indirect associations mediated by shared leaf‐miner hosts, comprised 22 host plant taxa and 33 parasitoid BINs, connected by 48 interaction types (65 interaction events; Figure 5a; Table S7). Network‐level specialization and modularity were significantly higher than expected under the null model (H2′ = 0.488, *z* = 7.67, *p* < 0.01; *Q* = 0.725, *z* = 5.09, *p* < 0.01; Table 2). Nestedness showed only a weak tendency to exceed null expectations, but the difference was not statistically significant (Table 2), whereas connectance was low and not significantly different from the null model.

The plant genera *Ayenia* (Malvaceae), *Nissolia* and *Lonchocarpus* (Fabaceae) acted as central nodes (Figure 5b), interacting with multiple parasitoids, predominantly from the parasitoid family Eulophidae. *Ayenia* hosted the highest diversity of eulophid parasitoids (10 BINs), with these associations largely mediated by the leaf‐mining moth *Atrotischeria* (BOLD:ACU3094, Lepidoptera: Tischeriidae). Other influential plant taxa included the genus *Guapira* (Nyctaginaceae) and the morphospecies Indet 1, each linked to two parasitoid interactions. Among parasitoids, two BINs stood out for their strong influence on overall network structure (Figure 5b): *Mirax* BOLD:AAU8805 (Hymenoptera: Braconidae) and BOLD:AFJ6717 (Hymenoptera: Eulophidae), both of which were associated with hosts feeding on plants from multiple families.

We identified 15 modules in the network (Figure 5c). To characterize indirect associations between parasitoids and plants, a PCA biplot based on Gini importance vectors (Figure 5d) revealed a primary gradient driven by host plant identity. This gradient separated modules associated with *Nissolia* and *Lonchocarpus* (Fabaceae) and Indet 1 from those structured by other plant lineages such as *Guapira* (Nyctaginaceae) and the eulophid parasitoid BOLD:AFJ3221 (Hymenoptera: Eulophidae). The second principal component (PC2) was dominated by parasitoid lineages with positive scores, in contrast to modules associated with *Ayenia* (Malvaceae).

### 3.8 Structure of the Tripartite Network

This tripartite network comprised 46 host plant taxa, 72 leafminer nodes, and 32 parasitoids connected through 116 interactions (Figure 6a, Table S4). The network exhibited high modularity (*Q* = 0.795), significantly greater than expected under the null model (*z* = 30.45, *p* < 0.001), indicating strong compartmentalization across trophic levels. This modular structure reflects the aggregation of tightly linked plant–leafminer–parasitoid subnetworks and is consistent with the high host specificity and structured host use observed in the bipartite networks (described below).

The network was partitioned into 33 modules (Figure 6a). Node‐importance analysis based on Mean Decrease Gini (MDG) for the 20 most influential taxa (Figure 6b) showed that 13 were host plants, with the remaining nodes comprising four leafminers and three parasitoids. Principal component analysis of Gini importance values (Figure 6c) revealed a gradient of interaction influence driven primarily by plant and herbivore nodes. *Nissolia* (Fabaceae) and BOLD:AFJ5665 (Coleoptera: Buprestidae) showed strong positive loadings on the first principal component, whereas parasitoid nodes such as BOLD:AFJ6717 (Eulophidae) and *Mirax* BOLD:AAU8805 (Hymenoptera: Braconidae) exhibited strong negative loadings. The second principal component was structured mainly by plant‐associated nodes, with *Apoplanesia* (Fabaceae) showing strong positive scores and *Acalypha* (Euphorbiaceae) strong negative scores.

### 3.9 Influence of Taxonomic Relatedness on Ecological Networks

Genetic relationships among BINs help explain the structure of modules and interactions in parasitoid networks. Mantel tests revealed a positive and significant correlation between genetic distance and Jaccard dissimilarity in both the parasitoid–leafminer network (*r* = 0.289, *p* = 0.001) and the parasitoid–plant network (*r* = 0.219, *p* = 0.003; Table 3).

**TABLE 3:** Relationship between genetic distance and interaction composition in the three bipartite networks. The Mantel test evaluated the association between genetic distances and ecological distances derived from the interaction matrices, while permutation tests compared the mean intra‐ and intermodular genetic distances according to module composition.

Network	Analysis	Observed	Null mean	Z‐score	Significance
Leaf miners–host plants	Mantel (*r*)	0.050	—	—	0.043*
	Intra‐module	0.22	0.23	−1.03	0.156
	Inter‐module	0.23	0.23	1.073	0.147
Parasitoids–leaf miners	Mantel (*r*)	0.29	—	—	0.001*
	Intra‐module	0.20	0.24	−2.89	0.009*
	Inter‐module	0.24	0.24	2.863	0.007*
Parasitoids–plants	Mantel (*r*)	0.21	—	—	0.003*
	Intra‐module	0.20	0.24	−2.94	0.005*
	Inter‐module	0.24	0.24	2.92	0.005*

*Note:* Significance values were estimated using 999 permutations. Asterisks indicate statistically significant results (*p* < 0.05).

Module structure further supported these patterns. In both parasitoid networks, observed mean genetic distances within modules were significantly lower than expected under the null model, whereas mean distances between modules were significantly higher (Table 3). These results indicate that interaction modules tend to group more closely related parasitoid taxa than expected by chance. In contrast, the leafminer–host plant network showed only a weak correlation between genetic distance and Jaccard dissimilarity (*r* = 0.049, *p* = 0.043), and mean genetic distances within and between modules did not differ significantly from null expectations (Table 3).

## 4 Discussion

### 4.1 Detecting Parasitoids From Immature Leaf‐Mining Insects

Our study highlights the value of high‐throughput DNA barcoding for characterizing leaf‐mining insect communities and their interactions with host plants and parasitoids in hyperdiverse tropical dry forests. Indeed, SMRT barcoding via PacBio proved particularly effective for detecting and identifying parasitoids living inside the leaf‐mining larvae/pupae based on secondary reads in the sample specific sequencing outputs. Parasitoid sequences were recovered from 12.8% of the sampled immature stages, illustrating the value of complementing traditional approaches, such as rearing (Lopez‐Vaamonde, Kirichenko, and Ohshima 2021), with molecular tools to capture the full diversity of parasitoid associations.

While high‐throughput sequencing has previously been used to resolve leaf miner‐parasitoid associations (Derocles et al. 2015), PacBio sequencing simplifies the process considerably compared with the pioneering studies of Kitson et al. (2019) and Nakadai and Kawakita (2017). Indeed, our approach enables hundreds to thousands of specimens to be multiplexed and sequenced simultaneously, reducing the effective effort per specimen (Sire et al. 2019), and per ecological interaction allowing the concurrent recovery of herbivore and parasitoid sequences from the same libraries, aligning with scalable high‐throughput barcoding approaches that multiplex large specimen sets in single runs (Hebert et al. 2018; Chua et al. 2023; Srivathsan et al. 2024). Crucially, our approach relies entirely on the standard CCDB high‐throughput DNA barcoding workflow, which typically targets ~250,000–350,000 CCS reads per run, without requiring multi‐locus or multi‐amplicon PCR. The only added step is downstream computational clustering and taxonomic assignment in mBRAVE. This means that multi‐trophic interaction networks can be generated within an established barcoding framework, facilitating adoption by laboratories already engaged in large‐scale barcode production. Although high‐throughput sequencing platforms are not yet equally accessible worldwide, the workflow builds on widely used barcoding infrastructure and maximizes the information recovered per specimen. This aligns with recent efforts to democratize biodiversity genomics through high‐throughput methods, notably the “reverse workflow” approach using Nanopore sequencing, which has been successfully applied to tropical insect surveys (Srivathsan et al. 2019, 2024; Meier et al. 2022).

### 4.2 Interaction Networks and Trophic Complexity

Our study highlights the complexity of ecological interactions in Chamela. From just 214 sequences, we identified 156 interaction types and 247 interaction events, demonstrating the power of molecular approaches to reveal hidden multi‐trophic interactions. The study also uncovered previously undocumented diversity, with over 57% of recovered BINs new to BOLD, including nearly all Coleopteran leafminers, emphasizing the high diversity of invertebrates in dry forest ecosystems. Importantly, the detection of parasitoid–host associations through molecular traces, without the need for rearing, represents a methodological advance, particularly in ecosystems where direct observations are logistically challenging.

Field collections were restricted to vegetation accessible from the ground and therefore primarily captured interactions associated with understory and shrub‐layer host plants. However, leafminers and their parasitoids are likely to interact with additional host plants and herbivores occurring in the canopy. Differences in leafminer community diversity across canopy strata have previously been reported in tropical dry forests (Medianero et al. 2003), and variation in the topology of leafminer interaction networks across canopy layers has also been documented (Chaij et al. 2016). Exploring canopy‐related differences in leafminer diversity will help us better understand the full extent of their diversity and the ecological processes shaping these insect communities.

The bipartite leaf miner‐plant network demonstrates low connectance and high specialization, where 58 BINs of leaf‐mining insects contributed to 67 interactions at the plant family level. This high degree of host specificity, with most leafminers relying on a single plant family, aligns with broader patterns in tropical ecosystems where guilds such as leafminers exhibit higher host fidelity than external feeders (Valladares and Salvo 2001; Novotny and Basset 2005). The top taxa based on Gini importance confirm this pattern, with plants rather than leafminers emerging as the most influential nodes. This suggests that the modular structure detected by the model is primarily driven by plant lineages rather than by individual leafminer taxa. In particular, the plant genera *Ayenia*, *Nissolia*, and *Guapira* appear to act as key nodes structuring interaction patterns within the network. Host plants contribute to differences in module composition, with distinct plant families characterizing different modules.

A recent study showed that plant–leafminer interaction networks exhibit broadly similar levels of nestedness, specialization, and modularity in Europe and North America (Chen et al. 2025). In contrast, our results reveal substantially higher levels of those metrics compared to those reported from these two continents (Table 2). This pattern is consistent with the expectation that biotic interactions are stronger in tropical systems, particularly in plant–insect herbivore systems where increased host specialization has been documented (Dyer et al. 2007; Forister et al. 2015).

Similarly, our plant–leafminer network exhibited higher modularity, network‐level specialization (H2′) and lower connectance than their associated parasitoid‐centered networks (Table 2). These differences could be explained because parasitoid–plant networks represent indirect, host‐mediated associations and therefore show less pronounced structure than networks describing direct trophic interactions. Together, these patterns are consistent with previous studies indicating that interaction structure weakens with increasing trophic distance from primary producers, while remaining constrained by the underlying plant–herbivore architecture (Lewinsohn et al. 2006; Thébault and Fontaine 2010). In this context, the presence of multiple eulophid genera known to include some hyperparasitoids (*Closterocerus*, *Chrysocharis*, *Elachertus*) further underscores the potential for higher‐order trophic interactions embedded within an otherwise plant‐mediated network structure, although confirmation of hyperparasitism would require targeted rearing or complementary molecular approaches.

When interpreted within a tri‐trophic framework, this strong plant–herbivore specificity forms the foundation upon which higher‐level interactions are structured, emphasizing the role of host plants as key organizers of multi‐trophic network architecture. Rather than acting independently, parasitoid associations emerge as constrained by herbivore–plant coupling, highlighting the importance of tri‐trophic linkage structure.

Braconidae and Eulophidae were the two dominant parasitoid families associated with leaf miners. Lepidoptera was the most common host group, accounting for over 60% of interactions in both parasitoid families. However, the frequency of interactions with Diptera and Coleoptera differed between the two families. Network topology was influenced by nodes from both trophic levels, suggesting that network structure reflects variation in both leaf miner‐parasitoid interactions and parasitoid host breath, with some parasitoid taxa showing more restricted (oligophagous) and others broader (polyphagous) interaction patterns.

Our approach was effective in revealing interactions that have not been previously documented. For example, this is the first report of a braconid parasitoid (BOLD:AAU8805, Mirax) associated with a coleopteran leafminer (Chrysomelidae), whereas hosts reported in the literature are typically lepidopteran leafminers (Salvo et al. 2011). This BIN was associated with two different leafminer families within Lepidoptera, despite parasitoids in this group often being considered highly specific at the family level (Eiseman 2026).

We also detected interactions involving OTUs assigned to the dipteran family Cecidomyiidae. Associations between cecidomyiids and leafminers have been previously reported (Barnes 1933; Gagné and Jaschhof 2021; Eiseman 2026). Although Cecidomyiidae is primarily composed of gall‐inducing insects, some species have occasionally been reared from leaf mines. The trophic nature of this association remains unclear but may involve predation by cecidomyiid larvae.

The presence of families such as Bethylidae, which can act as both parasitoids and predators, further highlights the complexity of the food web. By integrating bipartite and tripartite perspectives, our study contributes to a growing body of work demonstrating how tri‐trophic structure can be inferred from DNA‐based interaction networks and, to our knowledge, provides the first evidence suggesting potential hyperparasitoid involvement in leafminer systems based on non‐target molecular data.

### 4.3 Influence of Genetic Distance Relatedness on Ecological Networks

Taxonomic relatedness, approximated by genetic distances, contributed to structuring interaction patterns in parasitoid networks (Table 3). The positive correlations between genetic and ecological distances in both parasitoid–leafminer and parasitoid–plant networks indicate that more closely related parasitoids tend to exploit more similar sets of hosts and, indirectly, plant contexts. For instance, in the bipartite network between leafminers and parasitoids (Figure 4), module 9 is composed of seven species of eulophid parasitoids all attacking a tischeriid micromoth. This pattern is consistent with evolutionary conservatism in host use among parasitoids of leafminers (Lopez‐Vaamonde et al. 2005).

In contrast, the weak relationship observed in the leafminer–plant network, together with the lack of deviation from null expectations in module‐level analyses, suggests that modules do not contain more closely related leafminers than expected by chance and that host plants are associated with multiple leaf miner lineages. This contrasts with expectations of conserved host use in leafminers (Lopez‐Vaamonde et al. 2003; Chen et al. 2025) but may be explained by high taxonomic diversity of leafminers with members of different orders that exploit the same host plants. An additional factor that may contribute to the weak signal observed in the leafminer–plant network is the relatively low number of plant taxa represented in our dataset. Limited plant diversity can reduce variation in ecological distances and constrain the ability to detect patterns of taxonomic relatedness. Moreover, plant identification relied primarily on morphological traits, and many leaf mines were collected from plants that were not flowering at the time of sampling and therefore lacked diagnostic reproductive structures, limiting reliable taxonomic identification. In this context, the incorporation of molecular identification approaches for host plants (e.g., plant DNA barcoding) would improve taxonomic resolution and may reveal finer‐scale patterns of host use, thereby enhancing the detection of relationships between plant identity, herbivore associations, and network structure.

Nevertheless, some modules exhibit clear signals of conservatism. For instance, module 11 comprises three species of gelechiid micromoths all feeding on *Guapira* (Nyctaginaceae) (Figure 3), illustrating that phylogenetic structuring can still emerge locally despite an overall weak signal. Consistent with this, network partitioning and node‐importance analyses indicate that plant and herbivore taxa dominate overall network organization, whereas parasitoids contribute more locally within modules rather than shaping global interaction patterns.

Together, these results indicate that the influence of taxonomic relatedness on network structure varies across trophic levels and is shaped by both ecological context and interaction type, highlighting the complex interplay between evolutionary history and community assembly processes (Dormann et al. 2017).

### 4.4 Ecological Implications of Network Architecture

All three bipartite networks—leafminer–host plant, parasitoid–leafminer, and parasitoid–host plant—exhibited high modularity and low connectance, indicating a compartmentalized interaction structure in which species are organized into discrete modules (Table 2). The low nestedness observed across networks further suggests limited overlap between interactions, reinforcing a modular rather than nested architecture. The compartmentalization at the bipartite level is consistent with the high modularity detected in the integrated tripartite network, where plant–leafminer–parasitoid interactions are organized into 33 distinct multi‐trophic modules. In this context, tripartite modularity emerges from strong plant–herbivore specificity combined with structured parasitoid host use, rather than indicating system‐level robustness. The network properties describe the interaction structure but should be interpreted cautiously given the exploratory nature of the dataset and limited sampling, which may lead to underestimation of connectance and apparent inflation of modularity.

The high proportion of BINs not previously represented in BOLD, particularly within Coleoptera and Hymenoptera, highlights the still‐incomplete documentation of insect diversity in Mexican tropical dry forests. Thanks to extensive DNA barcoding efforts on Costa Rican insects, particularly in the tropical dry forest of Guanacaste (Janzen et al. 2009; Janzen and Hallwachs 2016), some of our Mexican BINs show geographic overlap with Costa Rica. However, many BINs appear to be locally restricted or remain unsampled elsewhere (Table S2). These patterns indicate substantial undocumented diversity and uneven taxonomic coverage across insect groups, even in relatively well‐studied systems such as leaf‐mining communities. Combined with the structured interaction networks described above, this hidden diversity suggests that both species richness and trophic interactions in tropical dry forests remain undercharacterized. Following the example of Costa Rica, expanding molecular reference libraries for the Mexican insect fauna will be critical to fully resolve species identities and interactions, thereby enhancing the accuracy and completeness of ecological network studies in these ecosystems.

## 5 Conclusions

A key contribution of this study is the demonstration that scalable, high‐throughput DNA barcoding of individual insects can effectively resolve multi‐trophic interactions without relying on labor‐intensive rearing. By enabling the detection of herbivore–parasitoid associations directly from individual immature stages (larvae and pupae), this approach provides high‐resolution mapping of ecological networks in poorly known environments. As molecular tools become increasingly accessible, DNA‐based interaction networks are poised to play a central role in comparative studies of trophic structure and in documenting biodiversity in complex ecosystems.

This study also characterizes the structural organization of leaf‐mining insect–parasitoid–host plant networks in tropical dry forests, highlighting the complexity of species associations across trophic levels. Observed network architecture, marked by high modularity, strong specialization, and low nestedness, indicates that interactions are organized into discrete plant–leafminer–parasitoid modules shaped by host specificity and structured parasitoid host use. Integration of bipartite and tripartite network analyses shows that tri‐trophic modularity emerges from strong plant–herbivore coupling combined with parasitoid segregation across host‐associated modules. These results underscore the central role of host plant identity in structuring lower trophic levels, while parasitoid interactions introduce additional, yet constrained, cross‐module linkages.

From a conservation perspective, our findings emphasize that protecting plant diversity in tropical dry forests is likely to be critical not only for preserving herbivore communities but also for maintaining higher trophic interactions structured around specific host lineages. Because many interactions were organized into discrete, plant‐centered modules, the loss of particular host plants could lead to the disappearance of associated herbivores and their parasitoids, with consequences for local interaction structure. Although further sampling is needed to assess the generality of these patterns, our results suggest that conserving habitat heterogeneity and host plant diversity will be important for sustaining multi‐trophic biodiversity in tropical dry forest ecosystems.

## Author Contributions

A.H.‐L., C.L.‐V., and A.Z.‐R. conceived the research; A.H.M.‐S. and A.H.‐L. participated in fieldwork; A.H.M.‐S. carried out leaf mine dissections and sample preparation for DNA barcoding; A.H.M.‐S., C.L.‐V., and A.H.‐L. performed data curation and data analysis; G.C.‐N. performed additional statistical and ecological analyses; R.T.C. identified the plant specimens; E.V.Z. produced sequencing results, including barcodes and coamplification data curation at the CCDB; A.H.M.‐S., A.H.‐L., and C.L.‐V. wrote the manuscript. All authors reviewed and approved the final version of the manuscript.

## Funding

This work was supported by Consejo Nacional de Ciencia y Tecnología, 58548; the European Union via the FEDER program FONDS EUROPEEN DE DEVELOPPEMENT REGIONAL – PROGRAMME OPERATIONNEL FEDER – FSE 2014–2020 CONSEIL REGIONAL CENTRE‐VAL DE LOIRE, SECIHTI, CBF 2025 G576.

## Disclosure

Benefit‐Sharing Statement: This research was conducted through a collaborative partnership between Mexican and international institutions, with scientists from the provider country (Mexico) involved at every stage of the project—from study design and fieldwork to data analysis and manuscript preparation. All collaborators from Mexico are included as co‐authors. The results have been shared with local stakeholders and the broader scientific community, including through public presentations and open‐access data deposited in the BOLD database (see above). This research contributes to national conservation priorities by enhancing knowledge of insect biodiversity and trophic interactions in Mexico's tropical dry forests—a critically endangered ecosystem. More broadly, our team is committed to equitable scientific partnerships, knowledge co‐production, and institutional capacity building, including the integration of students and early‐career researchers in biodiversity genomics. Fieldwork was carried out under official collection permits and in coordination with the Estación de Biología Chamela to ensure alignment with local conservation goals.

## Conflicts of Interest

The authors declare no conflicts of interest.

## Supporting information


**Figure S1:** Neighbour‐Joining tree of 244 sequences (project MEXIL). The seven sequences highlighted in blue correspond to pseudogenes.

**Table S1:** Metadata of the 284 records and 245 sequences (project DS‐LEAFCHAM).

**Table S2:** Summary statistics for 81 BINS (90 OTUs) included in this study.

**Table S3:** Co‐amplification of 31 non target sequences (NTS) and their associated hosts.

**Table S4:** Matrix of 116 tripartite interactions.

**Table S5:** Matrix of 118 bipartite interactions between leaf‐mining insects and their host plant.

**Table S6:** Matrix of 64 bipartite interactions between parasitoids and their host leafminers.

**Table S7:** Matrix of 65 bipartite interactions between parasitoids and hot plants.

## Data Availability

Metadata, including collection coordinates and voucher photographs of leaf mines and insects, have been deposited in the Barcode of Life Data Systems (BOLD) and are accessible under the dataset DS‐LEAFCHAM at https://doi.org/10.5883/DS‐LEAFCHAM. All sequence data are also available in GenBank.
