# Supplementary material for: High‐Throughput DNA Barcoding Reveals Multi‐Trophic Networks of Plants, Leafminers and Parasitoids in a Tropical Dry Forest
Source: Mol Ecol Resour. 2026 Aug 26;26(6):e70183. doi: 10.1111/1755-0998.70183 (PMC13509077; doi:10.1111/1755-0998.70183)
Supplement: Supplementary file 1 [file MEN-26-e70183-s003.pdf]

BOLD TaxonID Tree

Title	:	Tree Result - MEXIL (284 records selected)
Date	:	27-Jan-2026
Data Type	:	Nucleotide
Distance Model	:	Kimura 2 Parameter
Marker	:	COI-5P
Colourization	:	[blue]=Stop Codons [red]=Contamination or misidentification

Label	:	Sample ID
Label	:	Taxon
Label	:	Barcode Cluster (BIN)

Sequence Count	:	244
Species count	:	3
Genus count	:	15
Family count	:	21
Unidentified	:	234

BIN Count	:	81

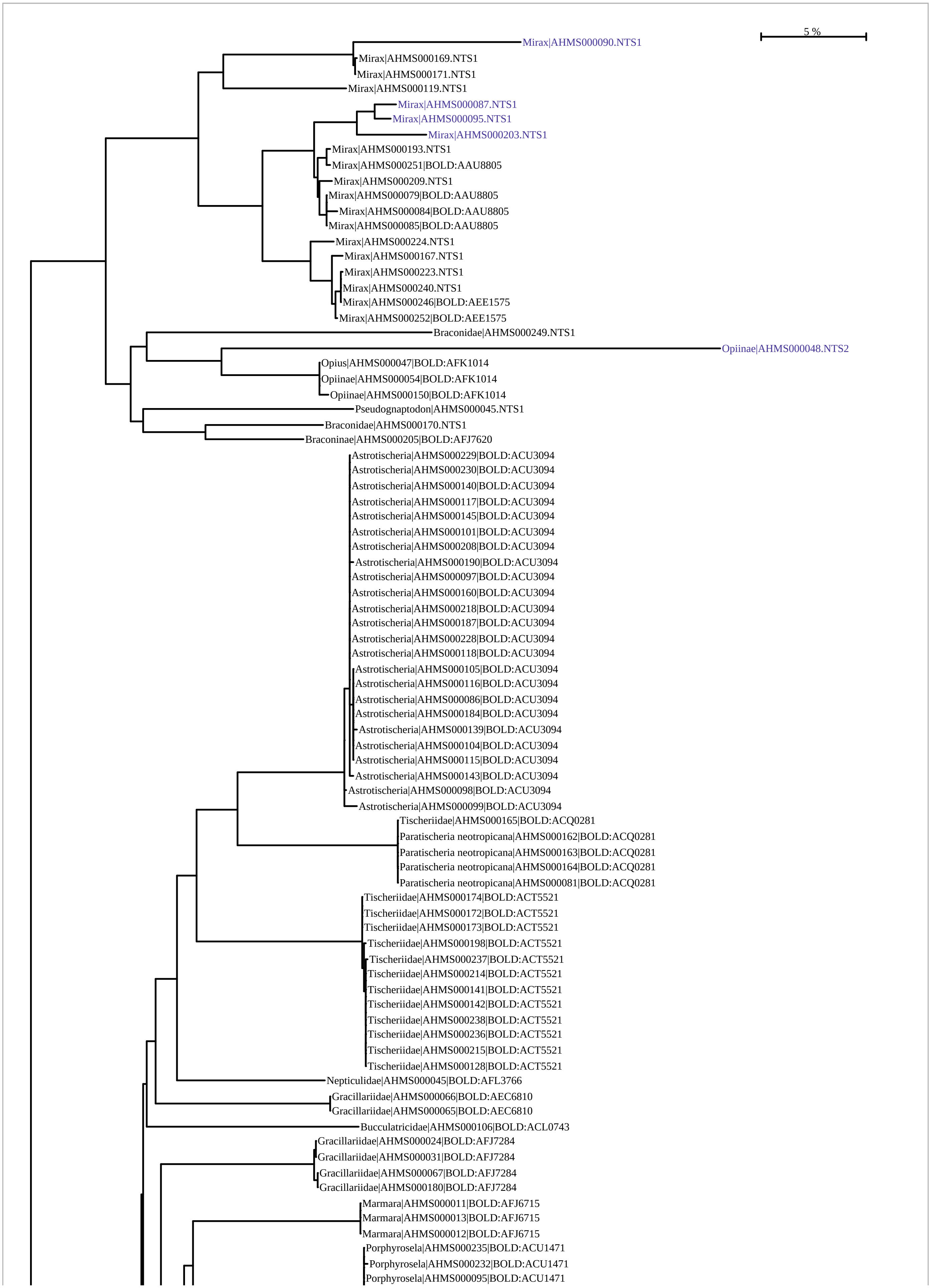
5 %
Mirax|AHMS000090.NTS1
Mirax|AHMS000169.NTS1
Mirax|AHMS000171.NTS1
Mirax|AHMS000119.NTS1
Mirax|AHMS000087.NTS1
Mirax|AHMS000095.NTS1
Mirax|AHMS000203.NTS1
Mirax|AHMS000193.NTS1
Mirax|AHMS000251|BOLD:AAU8805
Mirax|AHMS000209.NTS1
Mirax|AHMS000079|BOLD:AAU8805
Mirax|AHMS000084|BOLD:AAU8805
Mirax|AHMS000085|BOLD:AAU8805
Mirax|AHMS000224.NTS1
Mirax|AHMS000167.NTS1
Mirax|AHMS000223.NTS1
Mirax|AHMS000240.NTS1
Mirax|AHMS000246|BOLD:AEE1575
Mirax|AHMS000252|BOLD:AEE1575
Braconidae|AHMS000249.NTS1
Opiinae|AHMS000048.NTS2
Opius|AHMS000047|BOLD:AFK1014
Opiinae|AHMS000054|BOLD:AFK1014
Opiinae|AHMS000150|BOLD:AFK1014
Pseudognaptodon|AHMS000045.NTS1
Braconidae|AHMS000170.NTS1
Braconinae|AHMS000205|BOLD:AFJ7620
Astrotischeria|AHMS000229|BOLD:ACU3094
Astrotischeria|AHMS000230|BOLD:ACU3094
Astrotischeria|AHMS000140|BOLD:ACU3094
Astrotischeria|AHMS000117|BOLD:ACU3094
Astrotischeria|AHMS000145|BOLD:ACU3094
Astrotischeria|AHMS000101|BOLD:ACU3094
Astrotischeria|AHMS000208|BOLD:ACU3094
Astrotischeria|AHMS000190|BOLD:ACU3094
Astrotischeria|AHMS000097|BOLD:ACU3094
Astrotischeria|AHMS000160|BOLD:ACU3094
Astrotischeria|AHMS000218|BOLD:ACU3094
Astrotischeria|AHMS000187|BOLD:ACU3094
Astrotischeria|AHMS000228|BOLD:ACU3094
Astrotischeria|AHMS000118|BOLD:ACU3094
Astrotischeria|AHMS000105|BOLD:ACU3094
Astrotischeria|AHMS000116|BOLD:ACU3094
Astrotischeria|AHMS000086|BOLD:ACU3094
Astrotischeria|AHMS000184|BOLD:ACU3094
Astrotischeria|AHMS000139|BOLD:ACU3094
Astrotischeria|AHMS000104|BOLD:ACU3094
Astrotischeria|AHMS000115|BOLD:ACU3094
Astrotischeria|AHMS000143|BOLD:ACU3094
Astrotischeria|AHMS000098|BOLD:ACU3094
Astrotischeria|AHMS000099|BOLD:ACU3094
Tischeriidae|AHMS000165|BOLD:ACQ0281
Paratischeria neotropicana|AHMS000162|BOLD:ACQ0281
Paratischeria neotropicana|AHMS000163|BOLD:ACQ0281
Paratischeria neotropicana|AHMS000164|BOLD:ACQ0281
Paratischeria neotropicana|AHMS000081|BOLD:ACQ0281
Tischeriidae|AHMS000174|BOLD:ACT5521
Tischeriidae|AHMS000172|BOLD:ACT5521
Tischeriidae|AHMS000173|BOLD:ACT5521
Tischeriidae|AHMS000198|BOLD:ACT5521
Tischeriidae|AHMS000237|BOLD:ACT5521
Tischeriidae|AHMS000214|BOLD:ACT5521
Tischeriidae|AHMS000141|BOLD:ACT5521
Tischeriidae|AHMS000142|BOLD:ACT5521
Tischeriidae|AHMS000238|BOLD:ACT5521
Tischeriidae|AHMS000236|BOLD:ACT5521
Tischeriidae|AHMS000215|BOLD:ACT5521
Tischeriidae|AHMS000128|BOLD:ACT5521
Nepticulidae|AHMS000045|BOLD:AFL3766
Gracillariidae|AHMS000066|BOLD:AEC6810
Gracillariidae|AHMS000065|BOLD:AEC6810
Bucculatricidae|AHMS000106|BOLD:ACL0743
Gracillariidae|AHMS000024|BOLD:AFJ7284
Gracillariidae|AHMS000031|BOLD:AFJ7284
Gracillariidae|AHMS000067|BOLD:AFJ7284
Gracillariidae|AHMS000180|BOLD:AFJ7284
Marmara|AHMS000011|BOLD:AFJ6715
Marmara|AHMS000013|BOLD:AFJ6715
Marmara|AHMS000012|BOLD:AFJ6715
Porphyrosela|AHMS000235|BOLD:ACU1471
Porphyrosela|AHMS000232|BOLD:ACU1471
Porphyrosela|AHMS000095|BOLD:ACU1471

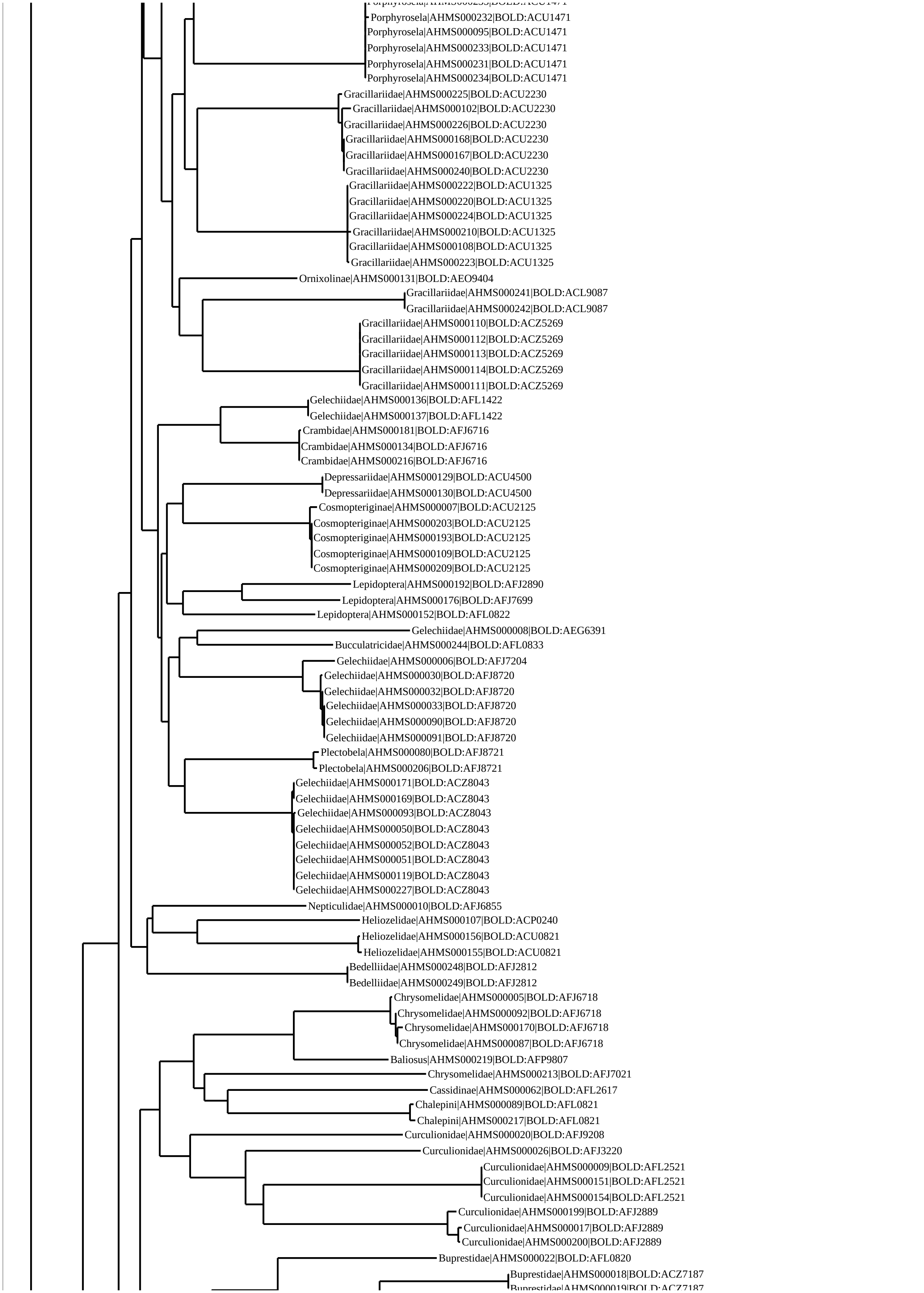
Porphyrosela|AHMS000232|BOLD:ACU1471
Porphyrosela|AHMS000095|BOLD:ACU1471
Porphyrosela|AHMS000233|BOLD:ACU1471
Porphyrosela|AHMS000231|BOLD:ACU1471
Porphyrosela|AHMS000234|BOLD:ACU1471
Gracillariidae|AHMS000225|BOLD:ACU2230
Gracillariidae|AHMS000102|BOLD:ACU2230
Gracillariidae|AHMS000226|BOLD:ACU2230
Gracillariidae|AHMS000168|BOLD:ACU2230
Gracillariidae|AHMS000167|BOLD:ACU2230
Gracillariidae|AHMS000240|BOLD:ACU2230
Gracillariidae|AHMS000222|BOLD:ACU1325
Gracillariidae|AHMS000220|BOLD:ACU1325
Gracillariidae|AHMS000224|BOLD:ACU1325
Gracillariidae|AHMS000210|BOLD:ACU1325
Gracillariidae|AHMS000108|BOLD:ACU1325
Gracillariidae|AHMS000223|BOLD:ACU1325
Ornixolinae|AHMS000131|BOLD:AEO9404
Gracillariidae|AHMS000241|BOLD:ACL9087
Gracillariidae|AHMS000242|BOLD:ACL9087
Gracillariidae|AHMS000110|BOLD:ACZ5269
Gracillariidae|AHMS000112|BOLD:ACZ5269
Gracillariidae|AHMS000113|BOLD:ACZ5269
Gracillariidae|AHMS000114|BOLD:ACZ5269
Gracillariidae|AHMS000111|BOLD:ACZ5269
Gelechiidae|AHMS000136|BOLD:AFL1422
Gelechiidae|AHMS000137|BOLD:AFL1422
Crambidae|AHMS000181|BOLD:AFJ6716
Crambidae|AHMS000134|BOLD:AFJ6716
Crambidae|AHMS000216|BOLD:AFJ6716
Depressariidae|AHMS000129|BOLD:ACU4500
Depressariidae|AHMS000130|BOLD:ACU4500
Cosmopteriginae|AHMS000007|BOLD:ACU2125
Cosmopteriginae|AHMS000203|BOLD:ACU2125
Cosmopteriginae|AHMS000193|BOLD:ACU2125
Cosmopteriginae|AHMS000109|BOLD:ACU2125
Cosmopteriginae|AHMS000209|BOLD:ACU2125
Lepidoptera|AHMS000192|BOLD:AFJ2890
Lepidoptera|AHMS000176|BOLD:AFJ7699
Lepidoptera|AHMS000152|BOLD:AFL0822
Gelechiidae|AHMS000008|BOLD:AEG6391
Bucculatricidae|AHMS000244|BOLD:AFL0833
Gelechiidae|AHMS000006|BOLD:AFJ7204
Gelechiidae|AHMS000030|BOLD:AFJ8720
Gelechiidae|AHMS000032|BOLD:AFJ8720
Gelechiidae|AHMS000033|BOLD:AFJ8720
Gelechiidae|AHMS000090|BOLD:AFJ8720
Gelechiidae|AHMS000091|BOLD:AFJ8720
Plectobela|AHMS000080|BOLD:AFJ8721
Plectobela|AHMS000206|BOLD:AFJ8721
Gelechiidae|AHMS000171|BOLD:ACZ8043
Gelechiidae|AHMS000169|BOLD:ACZ8043
Gelechiidae|AHMS000093|BOLD:ACZ8043
Gelechiidae|AHMS000050|BOLD:ACZ8043
Gelechiidae|AHMS000052|BOLD:ACZ8043
Gelechiidae|AHMS000051|BOLD:ACZ8043
Gelechiidae|AHMS000119|BOLD:ACZ8043
Gelechiidae|AHMS000227|BOLD:ACZ8043
Nepticulidae|AHMS000010|BOLD:AFJ6855
Heliozelidae|AHMS000107|BOLD:ACP0240
Heliozelidae|AHMS000156|BOLD:ACU0821
Heliozelidae|AHMS000155|BOLD:ACU0821
Bedelliidae|AHMS000248|BOLD:AFJ2812
Bedelliidae|AHMS000249|BOLD:AFJ2812
Chrysomelidae|AHMS000005|BOLD:AFJ6718
Chrysomelidae|AHMS000092|BOLD:AFJ6718
Chrysomelidae|AHMS000170|BOLD:AFJ6718
Chrysomelidae|AHMS000087|BOLD:AFJ6718
Baliosus|AHMS000219|BOLD:AFP9807
Chrysomelidae|AHMS000213|BOLD:AFJ7021
Cassidinae|AHMS000062|BOLD:AFL2617
Chalepini|AHMS000089|BOLD:AFL0821
Chalepini|AHMS000217|BOLD:AFL0821
Curculionidae|AHMS000020|BOLD:AFJ9208
Curculionidae|AHMS000026|BOLD:AFJ3220
Curculionidae|AHMS000009|BOLD:AFL2521
Curculionidae|AHMS000151|BOLD:AFL2521
Curculionidae|AHMS000154|BOLD:AFL2521
Curculionidae|AHMS000199|BOLD:AFJ2889
Curculionidae|AHMS000017|BOLD:AFJ2889
Curculionidae|AHMS000200|BOLD:AFJ2889
Buprestidae|AHMS000022|BOLD:AFL0820
Buprestidae|AHMS000018|BOLD:ACZ7187

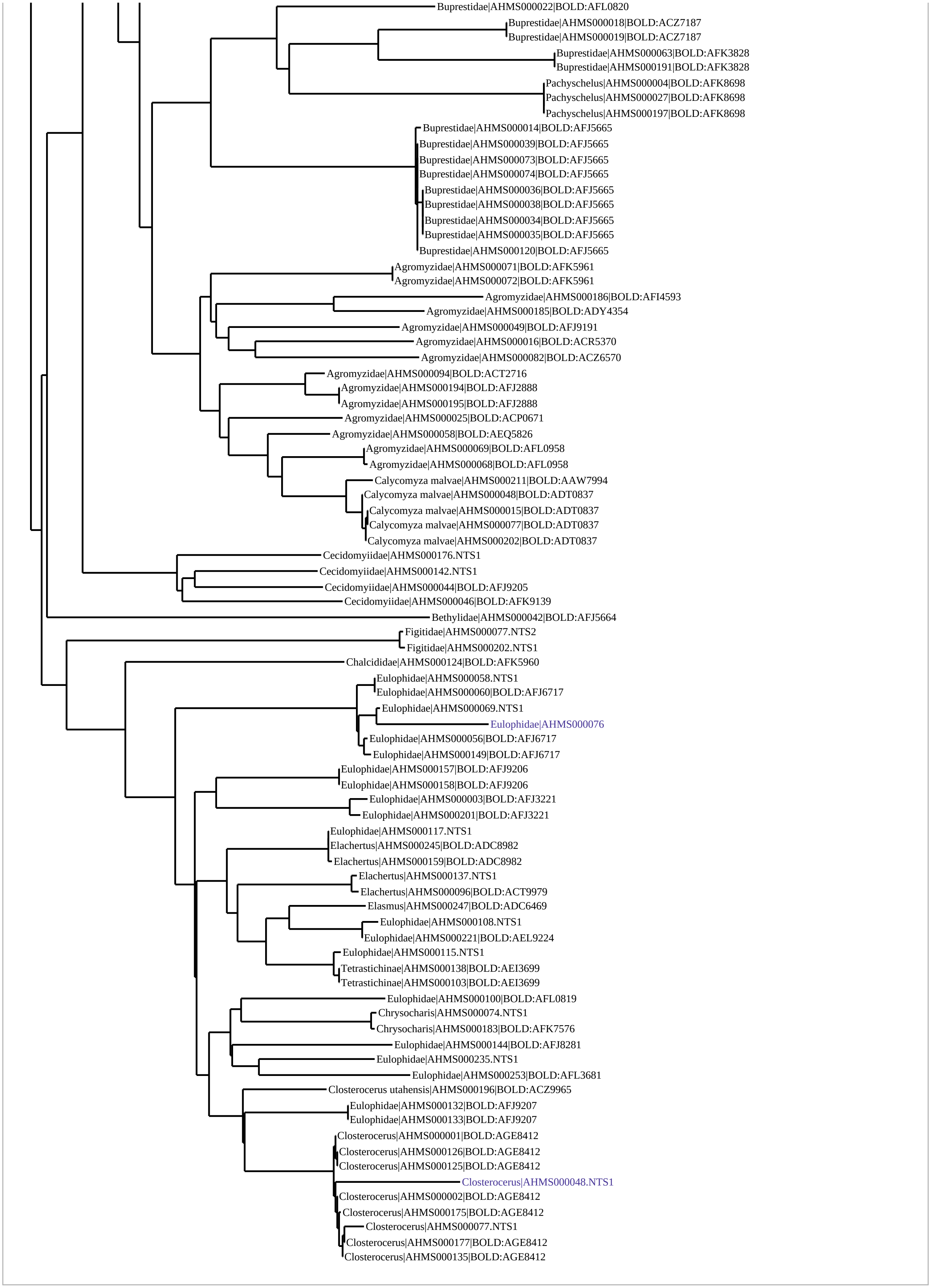
Buprestidae|AHMS000022|BOLD:AFL0820
Buprestidae|AHMS000018|BOLD:ACZ7187
Buprestidae|AHMS000019|BOLD:ACZ7187
Buprestidae|AHMS000063|BOLD:AFK3828
Buprestidae|AHMS000191|BOLD:AFK3828
Pachyschelus|AHMS000004|BOLD:AFK8698
Pachyschelus|AHMS000027|BOLD:AFK8698
Pachyschelus|AHMS000197|BOLD:AFK8698
Buprestidae|AHMS000014|BOLD:AFJ5665
Buprestidae|AHMS000039|BOLD:AFJ5665
Buprestidae|AHMS000073|BOLD:AFJ5665
Buprestidae|AHMS000074|BOLD:AFJ5665
Buprestidae|AHMS000036|BOLD:AFJ5665
Buprestidae|AHMS000038|BOLD:AFJ5665
Buprestidae|AHMS000034|BOLD:AFJ5665
Buprestidae|AHMS000035|BOLD:AFJ5665
Buprestidae|AHMS000120|BOLD:AFJ5665
Agromyzidae|AHMS000071|BOLD:AFK5961
Agromyzidae|AHMS000072|BOLD:AFK5961
Agromyzidae|AHMS000186|BOLD:AFI4593
Agromyzidae|AHMS000185|BOLD:ADY4354
Agromyzidae|AHMS000049|BOLD:AFJ9191
Agromyzidae|AHMS000016|BOLD:ACR5370
Agromyzidae|AHMS000082|BOLD:ACZ6570
Agromyzidae|AHMS000094|BOLD:ACT2716
Agromyzidae|AHMS000194|BOLD:AFJ2888
Agromyzidae|AHMS000195|BOLD:AFJ2888
Agromyzidae|AHMS000025|BOLD:ACP0671
Agromyzidae|AHMS000058|BOLD:AEQ5826
Agromyzidae|AHMS000069|BOLD:AFL0958
Agromyzidae|AHMS000068|BOLD:AFL0958
Calycomyza malvae|AHMS000211|BOLD:AAW7994
Calycomyza malvae|AHMS000048|BOLD:ADT0837
Calycomyza malvae|AHMS000015|BOLD:ADT0837
Calycomyza malvae|AHMS000077|BOLD:ADT0837
Calycomyza malvae|AHMS000202|BOLD:ADT0837
Cecidomyiidae|AHMS000176.NTS1
Cecidomyiidae|AHMS000142.NTS1
Cecidomyiidae|AHMS000044|BOLD:AFJ9205
Cecidomyiidae|AHMS000046|BOLD:AFK9139
Bethylidae|AHMS000042|BOLD:AFJ5664
Figitidae|AHMS000077.NTS2
Figitidae|AHMS000202.NTS1
Chalcididae|AHMS000124|BOLD:AFK5960
Eulophidae|AHMS000058.NTS1
Eulophidae|AHMS000060|BOLD:AFJ6717
Eulophidae|AHMS000069.NTS1
Eulophidae|AHMS000076
Eulophidae|AHMS000056|BOLD:AFJ6717
Eulophidae|AHMS000149|BOLD:AFJ6717
Eulophidae|AHMS000157|BOLD:AFJ9206
Eulophidae|AHMS000158|BOLD:AFJ9206
Eulophidae|AHMS000003|BOLD:AFJ3221
Eulophidae|AHMS000201|BOLD:AFJ3221
Eulophidae|AHMS000117.NTS1
Elachertus|AHMS000245|BOLD:ADC8982
Elachertus|AHMS000159|BOLD:ADC8982
Elachertus|AHMS000137.NTS1
Elachertus|AHMS000096|BOLD:ACT9979
Elasmus|AHMS000247|BOLD:ADC6469
Eulophidae|AHMS000108.NTS1
Eulophidae|AHMS000221|BOLD:AEL9224
Eulophidae|AHMS000115.NTS1
Tetrastichinae|AHMS000138|BOLD:AEI3699
Tetrastichinae|AHMS000103|BOLD:AEI3699
Eulophidae|AHMS000100|BOLD:AFL0819
Chrysocharis|AHMS000074.NTS1
Chrysocharis|AHMS000183|BOLD:AFK7576
Eulophidae|AHMS000144|BOLD:AFJ8281
Eulophidae|AHMS000235.NTS1
Eulophidae|AHMS000253|BOLD:AFL3681
Closterocerus utahensis|AHMS000196|BOLD:ACZ9965
Eulophidae|AHMS000132|BOLD:AFJ9207
Eulophidae|AHMS000133|BOLD:AFJ9207
Closterocerus|AHMS000001|BOLD:AGE8412
Closterocerus|AHMS000126|BOLD:AGE8412
Closterocerus|AHMS000125|BOLD:AGE8412
Closterocerus|AHMS000048.NTS1
Closterocerus|AHMS000002|BOLD:AGE8412
Closterocerus|AHMS000175|BOLD:AGE8412
Closterocerus|AHMS000077.NTS1
Closterocerus|AHMS000177|BOLD:AGE8412
Closterocerus|AHMS000135|BOLD:AGE8412
